# A Regulatory Code for Neuron-Specific Odor Receptor Expression

**DOI:** 10.1371/journal.pbio.0060125

**Published:** 2008-05-27

**Authors:** Anandasankar Ray, Wynand van der Goes van Naters, John R Carlson

**Affiliations:** Department of Molecular, Cellular, and Developmental Biology, Yale University, New Haven, Connecticut, United States of America; University of California, Berkeley, United States of America

## Abstract

Olfactory receptor neurons (ORNs) must select—from a large repertoire—which odor receptors to express. In *Drosophila*, most ORNs express one of 60 *Or* genes, and most *Or* genes are expressed in a single ORN class in a process that produces a stereotyped receptor-to-neuron map. The construction of this map poses a problem of receptor gene regulation that is remarkable in its dimension and about which little is known. By using a phylogenetic approach and the genome sequences of 12 *Drosophila* species, we systematically identified regulatory elements that are evolutionarily conserved and specific for individual *Or* genes of the maxillary palp. Genetic analysis of these elements supports a model in which each receptor gene contains a zip code, consisting of elements that act positively to promote expression in a subset of ORN classes, and elements that restrict expression to a single ORN class. We identified a transcription factor, *Scalloped*, that mediates repression. Some elements are used in other chemosensory organs, and some are conserved upstream of axon-guidance genes. Surprisingly, the odor response spectra and organization of maxillary palp ORNs have been extremely well-conserved for tens of millions of years, even though the amino acid sequences of the receptors are not highly conserved. These results, taken together, define the logic by which individual ORNs in the maxillary palp select which odor receptors to express.

## Author Summary

Odors are detected by olfactory receptor neurons (ORNs). Which odor an individual neuron detects is dictated by the odor receptors it expresses. Odor receptors are encoded by large families of genes, and an individual neuron must thus select the gene it expresses from among many possibilities. The mechanism underlying this choice is largely unknown. We have examined the problem of receptor gene choice in the fruit fly *Drosophila*, whose maxillary palp contains six functional classes of ORNs, each expressing different odor receptor genes. By comparing the DNA sequences flanking these genes in 12 different species of *Drosophila*, we have identified regulatory elements that are evolutionarily conserved and specific to each odor receptor. Genetic analysis of these elements showed that some act positively to dictate expression in a subset of ORNs, while others act negatively to restrict the expression of a receptor gene to a particular ORN class. We identified a transcription factor, Scalloped, that mediates repression. We were surprised to find that the odor response spectra of these neurons have been well-conserved for tens of millions of years, even though the amino acid sequences of their receptors have diverged considerably.

## Abstract 3

How does an olfactory receptor neuron select which odor receptor to express? A computational analysis of 12*Drosophila* genomes combined with mutational analysis identifies conserved*cis* elements and defines a regulatory code.

## Introduction

Odor discrimination is based on the differential activities of olfactory receptor neurons (ORNs), which in turn depend on the odor receptors that the ORNs express [1,2]. This raises an intriguing problem: how do individual ORNs select, from among a large repertoire, which receptor genes to express?

Two models—a deterministic model and a stochastic model—are often proposed to explain the problem of receptor gene choice [3]. In the deterministic model, different receptor genes contain different combinations of *cis*-acting elements, and an individual gene is selected in those ORNs with corresponding transcription factors. In the stochastic model, individual receptor genes are selected by an unknown, singular entity or process that can act on only one gene at a time.

In mammals, the expression of an individual receptor is restricted to a particular zone of the olfactory epithelium, but within a zone, the choice of one receptor by a neuron is widely believed to be accomplished via a stochastic mechanism, followed by negative-feedback inhibition [4–6]. Only a single allele of an OR gene is expressed in an ORN [7], a property that has recently been found to be widespread among 4,000 autosomal genes surveyed in the human genome [8]. A 2.1-kb region called the H element, defined by its high homology between human and mouse, was shown to be required for normal expression of several OR genes adjacent to it [4]. Further analysis of the H element suggested an elegant model in which it also acts as a *trans*-acting enhancer element that allows stochastic activation of a single OR gene in each neuron [5]; however, recent data have favored a model in which the primary function of the H region is to act in *cis*, as one of many *cis*-regulatory elements required for OR expression in the mouse [4,9,10]. These results focus attention on the question of how *cis*-regulation might underlie the strikingly sophisticated problem of receptor gene choice.

**Figure 1:**
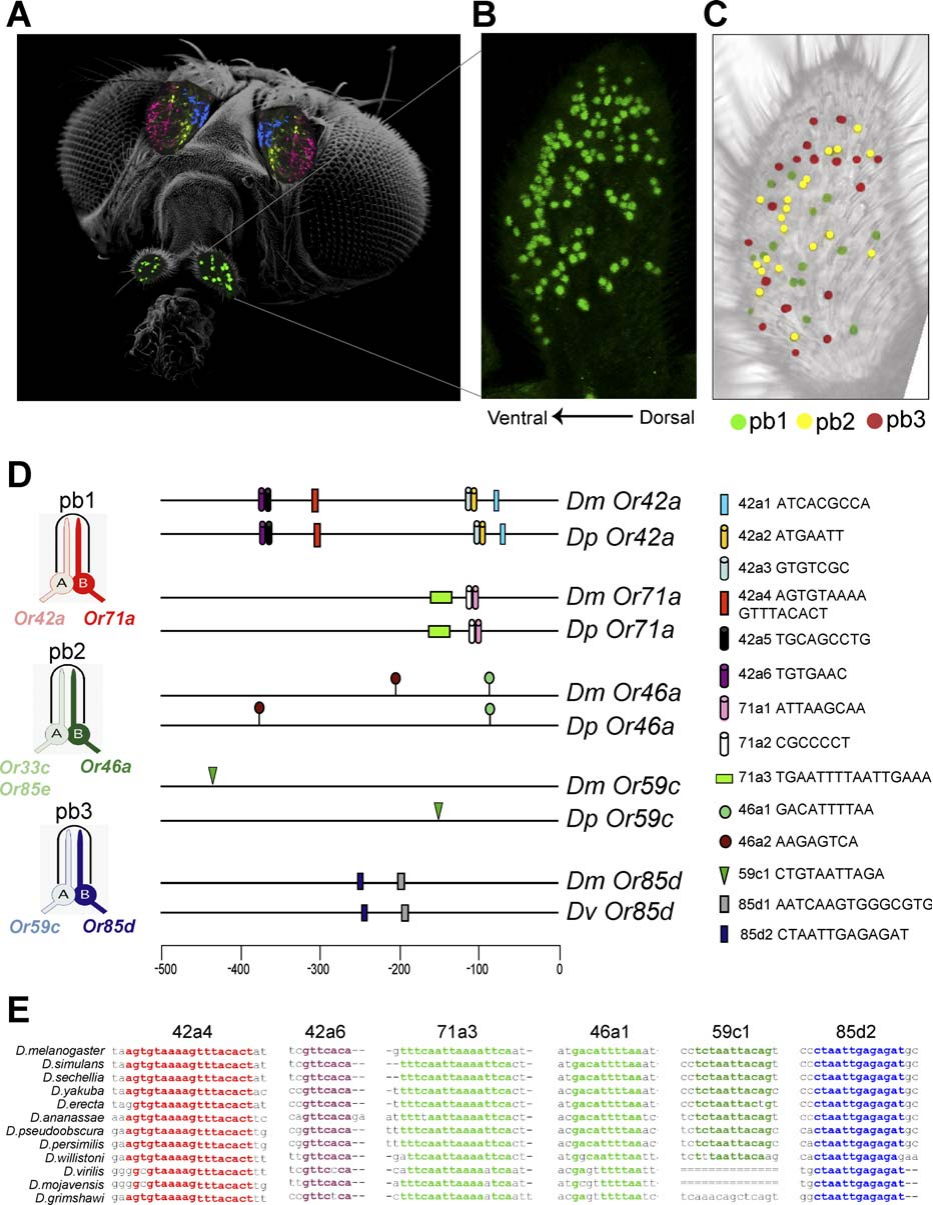
Maxillary Palp Organization and Conserved, Gene-Specific Elements (A) Representation of fly head, labeled with *Or22a-GAL4* (blue), *Or47a-GAL4* (yellow), *Or23a-GAL4* (magenta), and *Or71a-GAL4* (green) drivers, each used to drive *UAS-GFP*. The image is a merge of four different compressed Z-stacks of four different flies, with the GFP pseudo-colored differently for each receptor. The antenna is tricolored; the maxillary palp is green. (B) Nuclei of the ∼120 neurons of the maxillary palp, stained with anti-Elav antibody. (C) The three types of sensilla. The pb1, pb2, and pb3 sensilla are represented by green, yellow, and red respectively, as described in the Materials and Methods. (D) Map of the gene-specific conserved motifs in the upstream sequences of the maxillary palp *Or* genes. Names assigned to the different motifs include the unique part of the gene name and a number indicating relative proximity to the translation start site. (E) Sequence alignments of the most highly conserved motifs for each gene. In some cases the indicated sequences are the reverse complement of those shown in (D).

The fruit fly Drosophila melanogaster contains two olfactory organs, the antenna and the maxillary palp, each covered with olfactory sensilla (Figure 1A). Each sensillum contains ORNs, usually two, combined according to a strict pairing rule. In the antenna, each ORN class is restricted to a zone of the antennal surface, with zones showing varying degrees of overlap (Figure 1A and [11]). In the maxillary palp, physiological data showed that different types of sensilla, and by extension, different classes of ORNs, appear to be largely if not completely coextensive, as if the maxillary palp constituted a single zone [12].

There are 60 Odor receptor (*Or*) genes, most of which are expressed in either the antenna or the maxillary palp [13–16]. Each receptor is expressed in ORNs of a single functional class; ∼37 ORN classes have been defined [11,12,17–19]. Most ORN classes express a single receptor [19–22].

In an earlier study, we identified two regulatory elements that are required for organ-specific expression of receptor genes [23]. Within an organ, we found no evidence for a negative-feedback mechanism. However, we identified a *cis*- regulatory element required for receptor expression in one ORN class. These findings suggested the possibility that neuron-specific odor receptor choice in *Drosophila* may depend on a sophisticated combinatorial code of *cis-*regulatory elements, as opposed to a stochastic mechanism followed by a negative feedback mechanism. The results thus laid a foundation for a systematic investigation of the most challenging aspect of the problem: how different receptors are expressed in different ORNs of an individual organ. The maxillary palp was chosen because it offers the virtue of numerical simplicity. It contains ∼120 ORNs, which are housed in three types of sensillum: pb1, pb2, and pb3. Each sensillum contains two ORNs: pb1 contains pb1A and pb1B; pb2 contains pb2A and pb2B; pb3 contains pb3A and pb3B. The odor response profile of each ORN has been defined and a receptor-to-neuron map has been established [12,21]. Seven *Or* genes are expressed in the maxillary palp, with two genes coexpressed in the pb2A neuron.

We systematically identified novel regulatory elements that dictate the proper expression of the maxillary palp *Or* genes in the correct ORNs, that is, elements that underlie the receptor-to-neuron map. These elements were identified by using a phylogenetic approach, much as the H element was identified through a comparison of two species. We compared the regulatory regions of orthologs from two *Drosophila* species whose genomes have been sequenced, and we identified elements that are evolutionarily conserved and that are specific to individual maxillary palp *Or* genes. Analysis of these elements across all 12 sequenced *Drosophila* genomes identified six that are conserved particularly highly. Functional analysis of these six elements reveals that some act positively to express individual *Or* genes in a subset of ORNs, and some act negatively to restrict the expression of individual *Or* genes to a single ORN class. Repression can be mediated via upstream or downstream regions, and in one case is mediated by the transcription factor Scalloped. Some elements are also used in other chemosensory organs, and some are conserved upstream of genes required for ORN axon targeting, sorting, and guidance.

Taken together, the data support a model in which the receptor-to-neuron map is constructed via a system of molecular zip codes. *Or* genes contain three classes of regulatory elements: elements that specify expression in the correct organ, positive elements that activate *Or* genes in a subset of ORN classes within an organ, and negative elements that restrict expression to a unique ORN class within that organ. We propose that the concerted action of these three classes of elements thus solves a formidable biological regulatory problem. We carried out a functional analysis of the D. pseudoobscura maxillary palp. Surprisingly, we found a remarkable degree of conservation in the response spectra of the ORNs over tens of millions of years of evolution. The receptor-to-neuron map is also conserved.

## Results

### Spatial Overlap of ORN Classes in the Maxillary Palp

We examined the spatial organization of ORN classes in the maxillary palp. First, an anti-Elav antibody was used to illustrate the distribution of the entire population of ORN nuclei of the maxillary palp (Figure 1B). Second, we carried out a multiple-label experiment to differentially mark ORNs of the three types of sensilla: ORNs of the pb1A class were labeled in green, pb2B in yellow, and pb3A in red. The three classes of ORNs show extensive spatial overlap (Figure 1C). These results are consistent with the intermingling of sensillum types that are observed when recordings are taken from sensillar shafts [12]. The spatial overlap of ORN nuclei indicates that the identity of an ORN and, by extension, its choice of a receptor gene, are not dictated solely by its spatial position in a field.

### Identification of Conserved, Gene-Specific Elements

We previously compared the upstream regions of the two *Or* genes coexpressed in pb2A to identify regulatory sequences shared by these two genes, but not by any other maxillary palp *Or* gene [23]. To identify upstream regulatory elements for the other five maxillary palp *Or* genes, we used a different strategy based on phylogenetic analysis.

D. melanogaster and D. pseudoobscura diverged tens of millions of years ago [24] and contain orthologous receptor genes. We examined the upstream regions of orthologous *Or* genes for conserved elements shared by the members of each orthologous pair, but not by any of the other maxillary palp *Or* genes. Accordingly, we identified all conserved upstream sequences greater than 6 base pairs (bp) in length for each pair of orthologs using DOT-PLOT analysis (Figure S1A), and from these conserved elements we selected those that were specific to each gene. The analysis was focused on the 500 bp that are upstream of the translational start site, because in a previous study, this extent of DNA was sufficient to confer faithful expression to a *GAL4* reporter gene in the case of each of two maxillary palp *Or* genes analyzed in detail [23]. One pair of orthologs, *Or85d* and its D. pseudoobscura counterpart, was exceptionally well-conserved in the 500-bp upstream region, showing 80% identity. To identify discrete conserved elements within the region upstream of *Or85d*, we expanded our analysis to include a more divergent species, D. virilis.

Conserved, gene-specific elements were identified for each of the five *Or* genes analyzed (Figure 1D). The number of such elements varies: *Or59c* contains one, whereas *Or42a* contains six. In the special case of *Or85d*, two elements are shared by D. virilis and D. melanogaster upstream of *Or85d*, but are not found upstream of any other maxillary palp *Or* gene.

To identify the best candidate for a regulatory element for each of these receptor genes, we used a powerful bioinformatic approach that takes advantage of the recent sequencing of the genomes of ten other *Drosophila* species: D. simulans, *D. sechellia*, *D. yakuba*, *D. erecta*, *D. ananassae*, *D. persimilis*, *D. willistoni*, *D. virilis*, *D. mojavensis*, and D. grimshawi. The upstream regulatory regions of the orthologous receptor genes from all 12 species were aligned (Figure S1B) using the genome browser at the University of California Santa Cruz (http://genome.ucsc.edu/cgi-bin/hgGateway), and each of the elements was mapped onto the alignment. Using this approach, we were able to identify the gene-specific element with the highest sequence conservation for each of the receptor genes (Figures 1E and Figure S1); in the case of *Or42a*, two elements were nearly identical in their extent of conservation, and we have analyzed both.

### Gene-Specific Elements That Promote *Or* Expression

To determine whether the evolutionarily conserved, gene-specific elements have a regulatory function, we tested them in vivo using two complementary approaches, one based on a loss of function and one on a gain of function. For each gene, we analyzed the element with the highest sequence conservation. We did not analyze *Or85d* elements because we lacked a faithful *Or85d*-*GAL4* driver.

**Figure 2:**
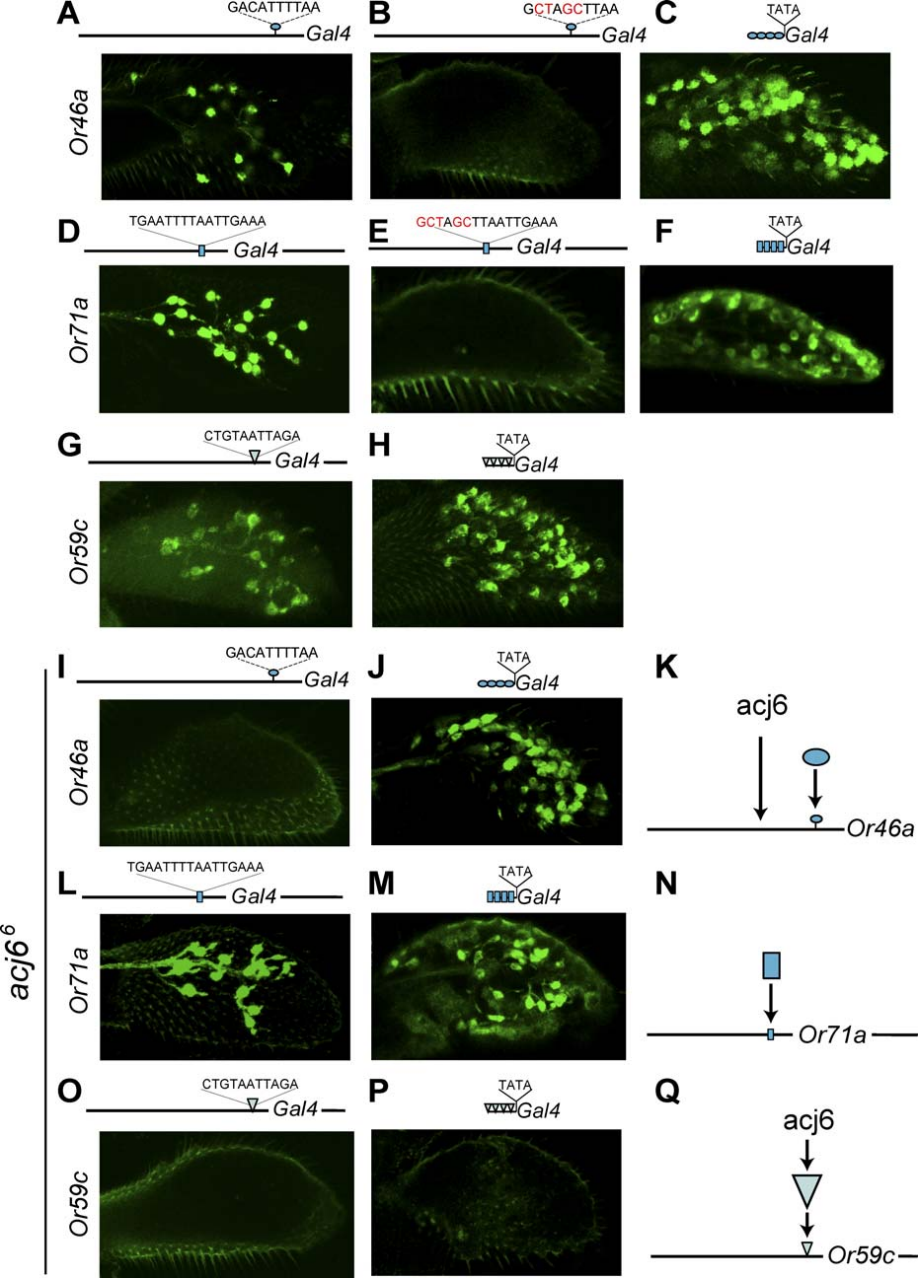
Functional Analysis of Gene-Specific Elements 46a1, 71a3, and 59c1 Expression of GFP driven by the wild-type promoter constructs (A, D, and G), mutated constructs (B and E), and minimal promoter constructs (C, F, and H). (A–C) 46a1; (D–F) 71a3; (G and H) 59c1. All flies contained one copy of the *Or-GAL4* constructs and two copies of *UAS-GFP*. At least eight independent transgenic lines were tested for each mutant construct, and at least two for each minimal promoter construct. Expression of GFP in an *acj6^6^* background driven by the wild-type *Or-GAL4* constructs (I, L, and O) and the minimal promoter constructs (J, M, and P). Dependence of elements on Acj6 (K, N, and Q). The arrow between acj6 and the large triangle in (Q) could reflect an effect on either expression or function. Images are Z-compressions of confocal stacks.

*Or46a* is expressed in the pb2B neuron, and its upstream region contains two conserved, gene-specific elements (Figures 1D and 1E and Figure S1). One of these elements, 46a1, is more highly conserved. It is 10 bp long, its sequence shows 93% identity across the 12 species, and its position is conserved. A 1.9-kb region of DNA upstream of *Or46a* drives faithful expression of a *GAL4* reporter in pb2B (Figure 2A and [21]). However, when the 46a1 element is mutated, the 1.9-kb region no longer drives expression (Figure 2B). In most cases, no cells are labeled; in rare cases, a single ORN is labeled (*n* = 0.52 ± 0.24 cells/maxillary palp; *n* = 8 independent lines examined; *n* > 10 maxillary palps examined per line). The simplest interpretation of these results is that the 46a1 element is necessary for *Or46a* expression in pb2B.

We then asked whether the 46a1 element can drive expression in the context of a minimal promoter. We placed four copies of 46a1 upstream of a TATA box and found that this small construct can in fact drive expression in maxillary palp cells (Figure 2C). Many, if not all, of the cells could be identified as ORNs, because they contain dendrites and axons; their identity is considered further below. Expression from this artificial promoter could also be detected in a small subset of neurons in the main gustatory organ, the labellum (unpublished data).

*Or71a* is expressed in pb1B. Its upstream region contains multiple gene-specific elements, of which the longest and best conserved is 71a3, consisting of 16 bp and showing 97% sequence identity. This element was tested in the context of the *Or71a* 5′ + 3′ construct, which contains sequences both upstream and downstream of *Or71a* [21]. This construct drives faithful expression of *GAL4* when the 71a3 element is intact (Figure 2D and [21]), but not when it is mutated (Figure 2E; *n* = 0.25 ± 0.1 cells/maxillary palp; *n* = 8 independent lines examined; *n* > 20 maxillary palps examined per line). When multiple copies of 71a3 were placed upstream of a TATA box, the construct drove *GAL4* expression in maxillary palp cells that can be identified as ORNs by virtue of their dendrites and axons (Figure 2F). Low levels of expression could also be detected in a small subset of cells in the labellum (unpublished data).

*Or59c* is expressed in pb3A, and its upstream region contains a single gene-specific conserved element, 59c1, which is 11 bp long and shows 97% sequence identity across nine species (Figure 2G); the region containing the 59c1 sequences could not be identified in three of the most distantly related species, D. virilis, *D. mojavensis* and D. grimshawi. We have tested its function by placing multiple copies upstream of a TATA box and found that this minimal promoter drove robust expression of GAL4 in the maxillary palp (Figure 2H). Expression was not detected in the labellum.

### Dependence of Neuron-Specific Elements on the POU Transcription Factor Acj6

Earlier studies have shown that the expression of a subset of the maxillary palp *Or* genes requires the POU domain transcription factor Acj6 [25], which is expressed in all ORNs of the maxillary palp [46]. Acj6 also controls axon targeting specificity of a subset of maxillary palp ORNs . The 46a1, 71a3, and 59c1 elements do not contain predicted Acj6 binding sites (Bai L, Carlson JR, unpublished results), and the transcription factors that act on these sequences are unknown. To test whether the factors that act on these neuron-specific elements are dependent on *acj6*, we examined the expression of the minimal promoter constructs in an *acj6^6^* background.

In the *acj6^6^* mutant, although the expression of the *Or46a-GAL4 driver* is lost, which is consistent with the loss of *Or46a* mRNA observed previously [13], the expression of the 46a1 minimal promoter construct is still strong (Figure 2I and 2J). These results suggest that the factors that direct expression from the 46a1 motif are independent of *acj6* for their expression and function (Figure 2K). An alternative possibility is that another transcription factor can compensate for the loss of *acj6*.

Expression of the *Or71a-GAL4* driver can be detected in *acj6*, and the expression of the 71a3 minimal promoter construct can also be detected (Figure 2L and 2M). These results suggest that the factors binding to 71a3 do not require *acj6* for their expression or function (Figure 2N).

In the case of *Or59c*, we find that *acj6* is required both for expression of the gene and for the minimal promoter (Figure 2O and 2P). These results suggest that *acj6* is required directly or indirectly for the expression of the 59c1 binding factor or for its function at the 59c1 site (Figure 2Q).

### A Gene-Specific Element That Represses *Or* Expression

*Or42a* is expressed in pb1A, and 4.1 kb of upstream DNA drives faithful expression of GAL4 in maxillary palp ORNs [21]. Two elements are nearly identical in their high conservation: 42a4 (98%) and 42a6 (98%), and we tested the function of both elements in vivo. 42a6 maps only three bp from 42a5 (Figure 1D). We constructed a small deletion that eliminates both 42a6 and 42a5 elements, and we found no effect on *Or42a*-*GAL4* expression (unpublished data).

**Figure 3:**
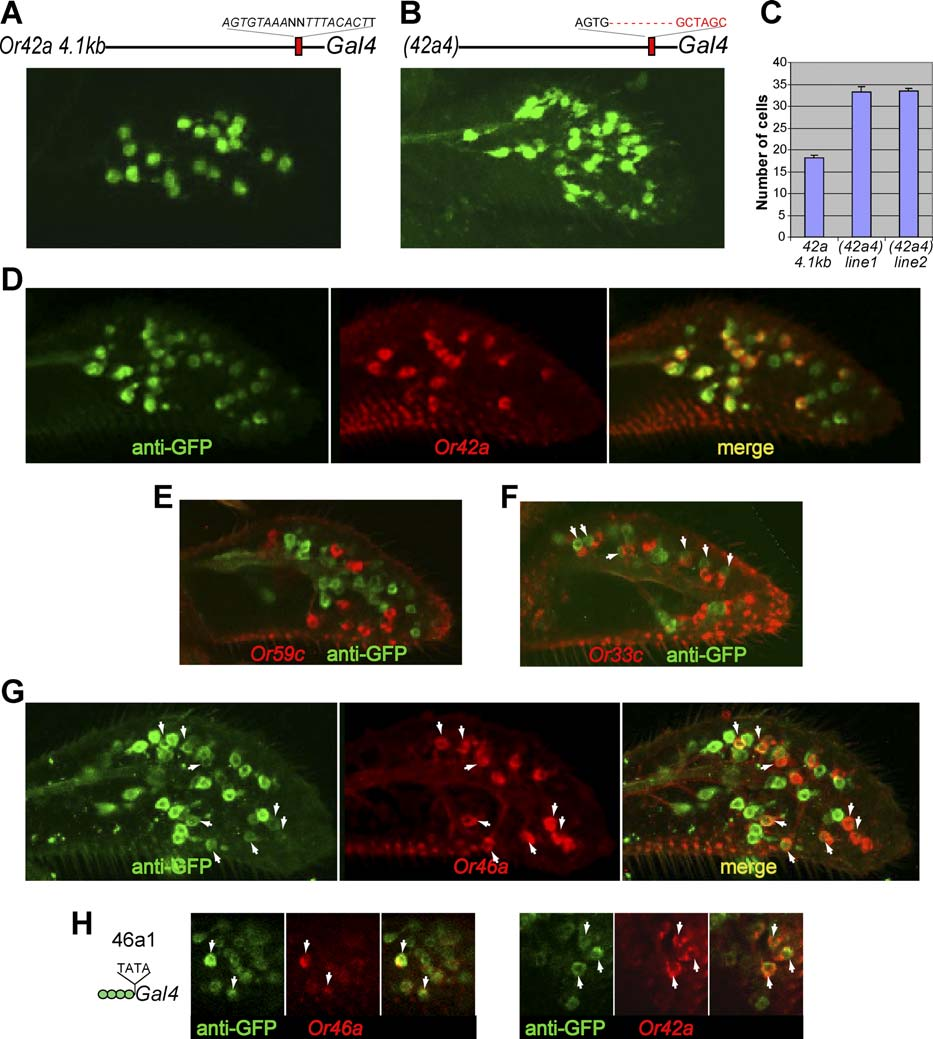
Neuron-Specific Repression Refines Expression of *Or42a* (A) Z-compression of confocal stacks showing expression of GFP driven by the wild-type *Or42a-GAL4* promoter; and (B) the same promoter with a mutation in the 42a4 element. (C) Numbers of GFP-positive cells per maxillary palp for the line shown in (A) and for two independent lines in which the 42a4 element is mutated; each value represents *n* = 9 maxillary palps. (D–G) Optical micrographs of (*42a4*)*-GAL4/UAS-GFP;UAS-GFP/+* maxillary palps labeled with anti-GFP antibody and the indicated RNA in situ hybridization probes. Arrowheads in (F) indicate paired cells; arrowheads in (G) indicate colabeled cells. (H) Optical sections of *46a1-GAL4/UAS-GFP;UAS-GFP/+* maxillary palps labeled with anti-GFP antibody and the indicated RNA in situ hybridization probes. Arrowheads indicate colabeled cells.

The longer of the two most highly conserved elements at *Or42a*, 42a4, contains an inverted repeat: AGTGTAAAAGTTTACACTT. We were surprised to find that mutation of this element led to a 2-fold increase in the number of labeled maxillary palp cells, from 18.2 ± 1.8 (*n* = 9 maxillary palps) to 33.2 ± 3.7 (*n* = 9 maxillary palps quantified from two independent lines; *n* = 8 independent lines examined, *n* > 20 maxillary palps examined/line) (Figure 3A–3C). The simplest interpretation of this result is that 42a4 is a negative regulatory element that represses *Or42a* in a subset of ORNs. To test this interpretation, we first carried out a double-label experiment using probes for the endogenous *Or42a* mRNA and for the green fluorescent protein (GFP) that is driven by the mutant promoter via GAL4. We found that all *Or42a^+^* cells express GFP, but that GFP is also expressed in an additional subset of cells (Figure 3D).

To identify the cells that ectopically express GFP, we undertook a series of additional double-label experiments. We found that the GFP^+^ cells do not express *Or59c* mRNA, indicating that they are not pb3A neurons (Figure 3E; 0% of the GFP^+^ neurons are *Or59c^+^*; *n* = 8 maxillary palps); nor are they paired with cells that express *Or59c* mRNA, indicating that they are not pb3B neurons. In another experiment, GFP^+^ cells did not label with an *Or33c* probe (only 3% of the GFP^+^ neurons appear *Or33c^+^*; *n* = 8 maxillary palps), indicating that they are not pb2A neurons; however, GFP^+^ cells were often found paired with *Or33c^+^* cells (arrowheads), indicating that many GFP^+^ cells are pb2B neurons (Figure 3F). The identity of these GFP^+^ cells as pb2B neurons was confirmed directly in another double-label experiment using a probe for *Or46a* mRNA (Figure 3G; 94% of the cells labeled with *Or46a* mRNA were GFP^+^; this value is the mean of values determined from *n* = 8 maxillary palps).

The simplest interpretation of these results is that positive regulatory elements in the *Or42a* upstream region are capable of driving expression not only in the pb1A neuron but also in the pb2B neuron. The 42a4 element represses expression in pb2B neurons, thereby restricting expression to a single ORN class, pb1A.

### Reciprocal Control of *Or42a* and *Or46a*

The ectopic expression of an *Or42a* promoter in *Or46a^+^* neurons suggested a relationship between these two genes. Further evidence for a relationship came from analysis of the minimal promoter containing multiple copies of 46a1 (Figure 2C). This promoter drove GFP expression in more ORNs than could be accounted for by *Or46a^+^* neurons alone. A double-label experiment showed that while most of the GFP^+^ cells are in fact *Or46a^+^*, some are *Or42a^+^* (Figure 3H).

The reciprocal relationship between *Or42a* and *Or46a* misexpression suggests that *Or42a* may contain an unidentified positive regulatory element, 42ax, that is similar in sequence to 46a1, with both sites able to bind a transcription factor present in both pb1A and pb2B. To test this interpretation, we examined the 500 bp upstream region of *Or42a* for an element similar, but not identical, to 46a1 (GACATTTTAA). We identified a sequence, TATATTTTAA, identical to 46a1 at the 8 underlined positions, at −455 bp. Moreover, these two sequences share an ATTTTA core, which has been shown to function as a binding site for basic helix-loop-helix transcription factors at other loci. TATATTTTAA is not found upstream of any other maxillary palp *Or* genes. This 42ax sequence is conserved in sequence (80% identity) and location in seven of the 12 *Drosophila* species. It will be interesting to identify the transcription factor that binds 46a1 and then test directly its binding to 42ax.

### Expression of *Or59c* Is Refined by Repression via Downstream Sequences

**Figure 4:**
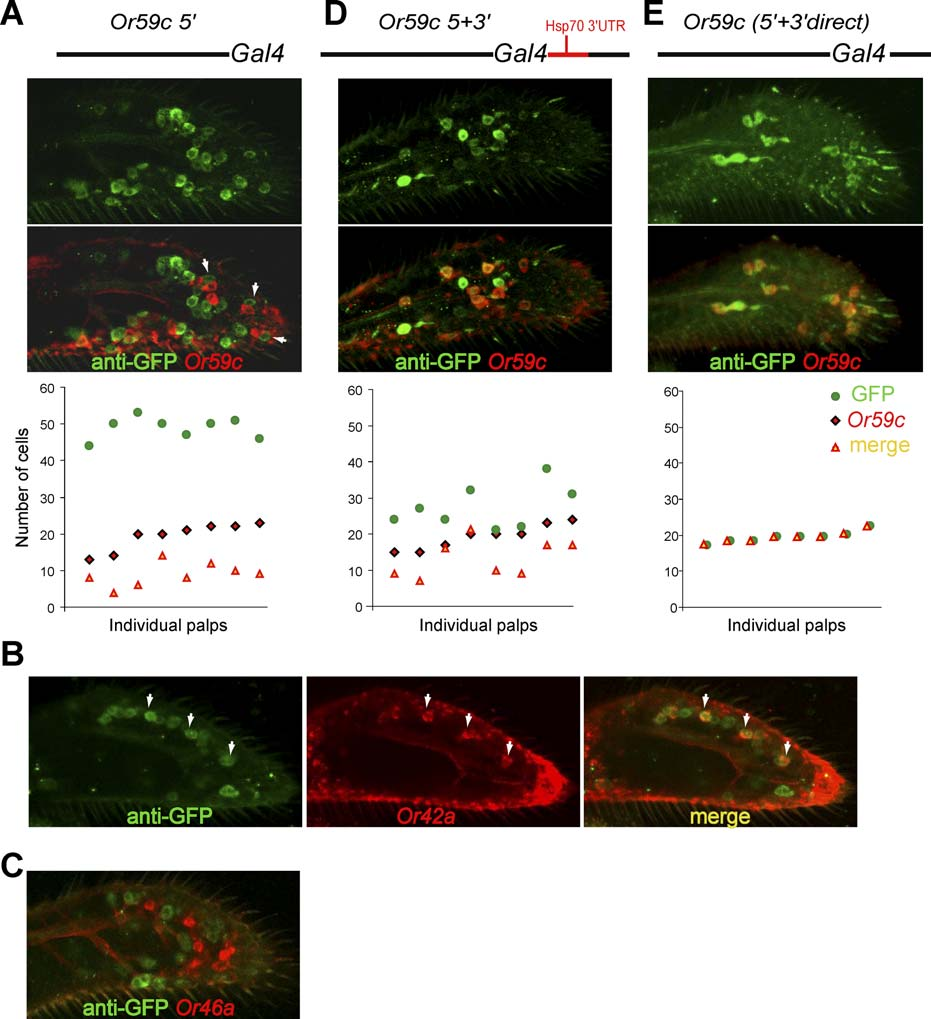
Neuron-Specific Repression of *Or59c* Acts via the Downstream Region (A, D, and E) Confocal micrographs showing cells labeled by anti-GFP antibody (top) driven by the indicated flanking sequences. The corresponding middle panels show double-labeling with an anti-GFP antibody and an *Or59c* RNA in situ hybridization probe. Graphs (below) indicate the numbers of GFP^+^ cells, *Or59c* ^+^ cells, and GFP^+^ *Or59c* ^+^ cells, for eight individual maxillary palps of each genotype. For each palp, the three indicated values are plotted in a vertical column. Each arrowhead in (A) indicates labeling of paired cells in a sensillum. (B andC) Optical sections from *59c5′-GAL4/UAS-GFP;UAS-GFP/+* maxillary palps labeled with an anti-GFP antibody and the indicated RNA probes.

When DNA upstream of *Or59c* was fused to *GAL4*, expression of the reporter GFP was not faithful (Figure 4A; *n* = 5 independent lines); the same result was obtained when upstream regions of varying lengths were used (either 2.1 kb, which extends to the next upstream gene, or 5.2 kb, which includes upstream coding sequences). Double-label experiments using an *Or59c* probe revealed misexpression in many *Or59c^–^* cells; moreover, many *Or59c^+^* cells did not express GFP. Some of the misexpressing cells are the neighboring pb3B neurons, which can be seen to be paired with *Or59c^+^* pb3A cells (arrowheads in Figure 4A; 75% of the cells neighboring the *Or59c^+^* cells were GFP^+^, *n* = 9 palps). To identify the other ORNs that ectopically express the *Or59c-GAL4* construct, we carried out double-label experiments with other *Or* genes. Misexpression was also observed in pb1A cells, which express *Or42a* (96% of the *Or42a^+^* cells misexpressed GFP, *n* = 9 palps), but not in the pb1B cells (Figure 4B), nor in the pb2A or B cells (Figure 4C). In summary, misexpression is specific to pb1A and pb3B.

Because neither of the varying lengths of upstream DNA sequences were sufficient to restrict *GAL4* expression to the *Or59c^+^* cells, we added 3′ sequences to the construct. Initially, 500 bp of DNA taken directly from the region immediately downstream from the *Or59c* stop codon was added downstream of the *GAL4* coding region. Between the downstream sequences of *Or59c* and the *GAL4* coding region was the *Hsp70* 3′ untranslated region (UTR), which is present in the *GAL4* vector and which is often present in promoter-*GAL4* analysis.

This *Or59c* 5′ + 3′ construct showed much less misexpression in *Or59c* ^−^ cells (Figure 4D). The total number of GFP^+^ cells declined from 49.7 ± 1.3 to 27.3 ± 2.1 (SEM; *n* = 10 in each case). However, some misexpression remained, and only 62% of the *Or59c^+^* neurons were GFP^+^. We then removed the *Hsp70* 3′ UTR sequences, such that the *Or59c* downstream sequences were in close proximity to the 3′ end of the *GAL4* coding region and the *Or59c* 3′ UTR is used. This construct drove faithful expression (Figure 4E; *n* = 8 independent lines examined). Thus, there is a negative regulatory element downstream of *Or59c* that restricts expression of this gene to pb3A neurons, and either there is a requirement that the native 3′ UTR be used, or else there is a regulatory factor that acts on this element in a context-dependent fashion in order to achieve this negative regulation. We note with interest that the inclusion of the downstream sequences, without the Hsp70 sequences, also drove expression in *Or59c^+^* neurons that had previously failed to express the reporter, suggesting that the downstream sequences are required for positive as well as negative regulation of *Or59c*.

### *scalloped* Represses *Or59c* in the Neighboring Neuron

**Figure 5:**
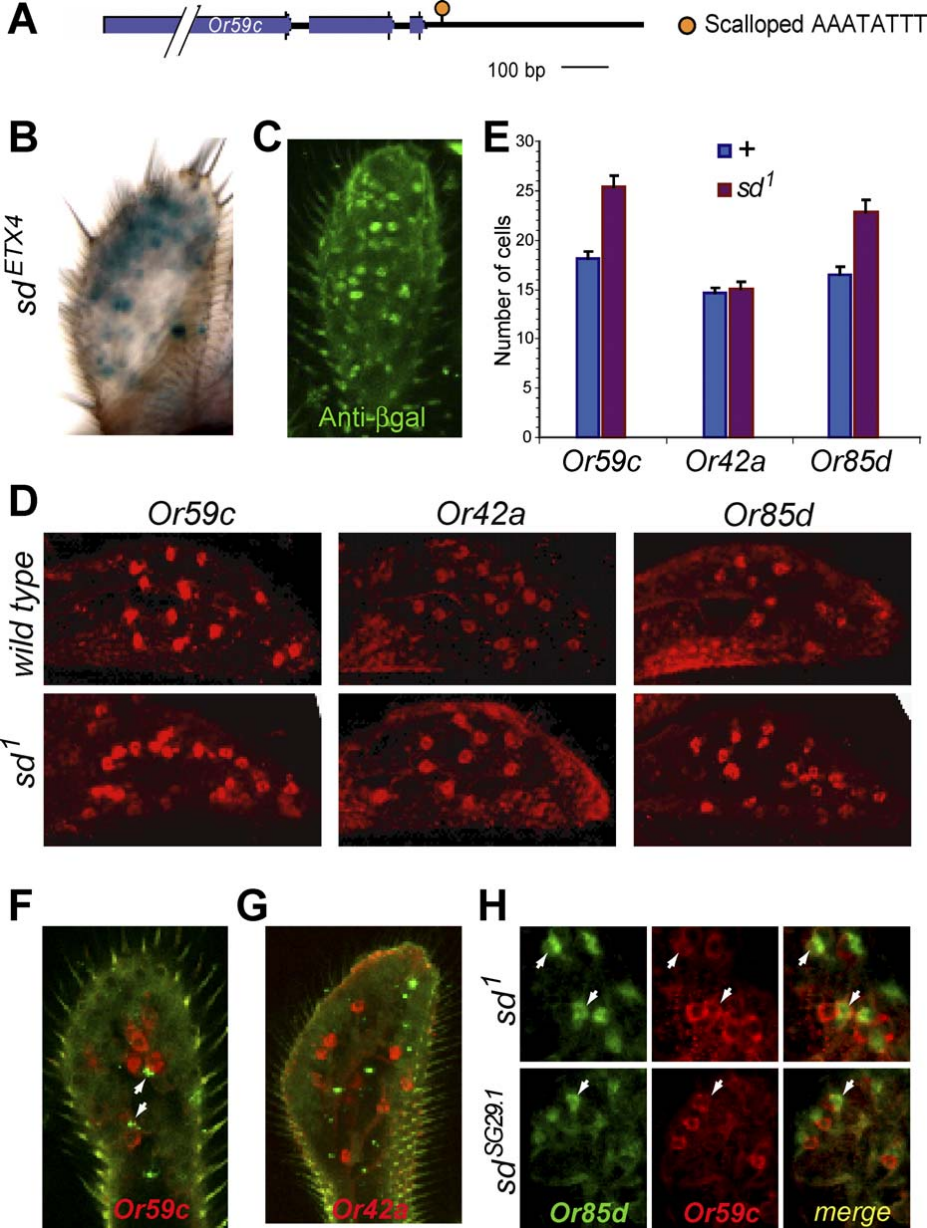
Scalloped-Mediated Repression of *Or59c* (A) Predicted Sd binding site downstream of *Or59c*. (B and C) Expression of a *sd* enhancer trap line, *sd^ETX4^*, visualized by X-gal staining (B) and anti-βGAL antibody staining (C). (D) RNA in situ hybridizations to maxillary palps of wild type and *sd^1^*, a hypomorphic allele. (E) Numbers of *Or RNA* positive cells per maxillary palp (*n* = 10; SEM). (F and G) Optical sections of *sd^ETX4^* maxillary palps labeled with anti-βGAL antibody (green) and RNA probes (red) for *Or59c* (F) and *Or42a* (G)*.* Arrowheads in (F) indicate βGAL^+^ cells paired with *Or59c^+^* cells. (H) Optical sections of double-label in situ hybridizations to maxillary palps of two *sd* mutants. Arrowheads indicate *Or85d^+^Or59c^+^* cells.

Inspection of the sequences downstream of *Or59c* that repressed misexpression revealed a binding site for the transcription factor Scalloped (Sd), AAATATTT [26] (Figure 5A). This site is well-conserved among a number of other species (Figure S2A). *Sd* has been shown to be expressed in olfactory organs [27]. To confirm and extend the description of *sd* expression we used an enhancer trap line, *sd^ETX4^* [27], and confirmed that *sd* is expressed in a subset of cells in the maxillary palp (Figure 5B and 5C).

To test whether *sd* represses *Or59c*, we carried out in situ hybridizations to the maxillary palp of a hypomorphic *sd* mutant, *sd^1^* (Figure 5D). We found a 40% increase in the number of *Or59c^+^* neurons (Figure 5E). By contrast, there was no increase in the number of *Or42a* ^+^ neurons (Figure 5D and 5E). There was, however, an increase in the number of *Or85d^+^* cells, and we note with interest that there is another type of Sd binding site, TAAAATTA [26], 737 bp downstream from the stop codon of *Or85d*.

The *Or59c-GAL4* construct that contains only upstream sequences, *Or59c* 5′, misexpresses in two ORN classes, the neighboring pb3B cell (*Or85d^+^*) and pb1A (*Or42a^+^*), as shown above in Figure 4. We asked whether *sd* is expressed in these two ORN classes. Using an *Or59c* probe, which labels the pb3A cell, we found that *sd* is in fact expressed in neighboring cells (Figure 5F), but not in pb1A cells, which express *Or42a* (Figure 5G). These results suggest that Sd may repress the *Or59c* gene in pb3B. If so, we would expect that in an *sd* mutant, we would observe cells that coexpress *Or59c* and *Or85d*. We tested this possibility by carrying out double-label in situ hybridizations in two different hypomorphic alleles of *sd*, *sd^1^*, and *sd^SG29.1^* [28]. In both alleles, we found *Or59c^+^ Or85d^+^* cells (Figure 5H), but not *Or59c^+^ Or42a^+^* cells (unpublished data). Thus repression of *Or59c* in the neighboring pb3B cell requires both a Sd binding site and Sd.

Since Sd represses *Or59c* in pb3B, why doesn't Sd also repress *Or85d* in pb3B, given that both *Or* genes have Sd binding sites? The simplest explanation is that the two Sd binding sites are distinct. There are several potential interacting partners with which Sd may interact to form a functional transcription factor [26,29], and the pb3B cell may contain a partner necessary for repression at the *Or59c* binding site but not a partner necessary for repression at the *Or85d* binding site. If a faithful *Or85d-GAL4* construct becomes available, it will be interesting to replace the *Or85d*-type Sd binding site with the *Or59c*-type Sd binding site, to determine whether the *Or59c*-type site confers repression in the pb3B cell.

We note that *Or85d*-*GAL4* constructs containing only the 5′ regions of *Or85d*, which lack the Sd binding site, drive misexpression in a number of non-neuronal cells of the maxillary palp (Figure S2B). Most of the labeled cells lack dendrites and axons, and when labeled with a membrane-bound GFP, as opposed to with RNA probes that label the cell bodies, these cells appear larger than ORNs. These results suggest that Sd may interact with a binding partner in non-neuronal cells to repress *Or85d* expression in these cells.

### Mechanisms of Receptor Gene Choice in the Maxillary Palp Are Used Elsewhere

**Figure 6:**
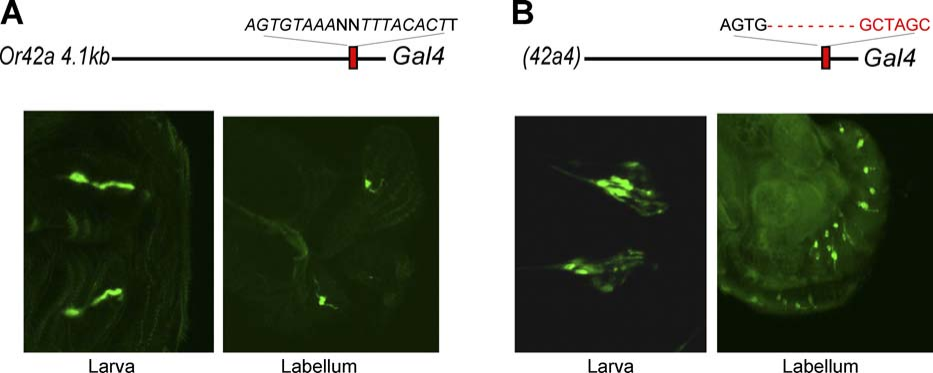
Conservation of Regulatory Logic in Other Systems Confocal micrographs showing expression of GFP driven by the wild-type *Or42a* promoter (A) and a mutant (*42a4)-GAL4* promoter (B) in larval olfactory neurons and the adult labellum.

*Or42a* is expressed in the larval olfactory system as well as in the maxillary palp [21,30]. The *Or42a-GAL4* construct shows expression in one ORN in each of the bilaterally symmetric larval olfactory organs, the dorsal organs (Figure 6A). We also observed expression in two neurons of the labellum, the taste organ on the adult head (Figure 6A). To determine whether the conserved elements identified in our analysis of maxillary palp receptor choice can act in these other chemosensory organs, we examined *Or42a-GAL4* constructs in which these elements were mutated. A mutation that affects both 42a6 and 42a5, which did not affect expression in the maxillary palp, had no effect on expression in these other organs. However, mutation of 42a4, which relieved repression of *Or42a* in other maxillary palp ORNs, also relieved repression of *Or42a-GAL4* in the larval olfactory organs and the labellum (Figure 6B): in both cases supernumerary neurons were labeled. In the labellum, ∼8–10 pairs of neurons were labeled. These results suggest that the molecular mechanisms underlying receptor gene choice in the maxillary palp overlap with those specifying receptor expression in other chemosensory organs.

### The Response Spectra and Organization of Maxillary Palp ORNs Have Been Conserved for Tens of Millions of Years

In this study we have identified and functionally characterized a number of regulatory elements that operate in directing the formation of the receptor-to-neuron map of D. melanogaster. Because the newly defined elements we have analyzed here are conserved in sequence and position among *Drosophila* species, we predicted that the programmed regulation leading to the formation of receptor-to-neuron maps would be conserved as well. To test this prediction, we carried out a physiological analysis of the D. pseudoobscura maxillary palp. Although each of the seven *Or* genes expressed in the maxillary palp has an ortholog expressed in the D. pseudoobscura maxillary palp ( as described [21] and unpublished data), we expected that their odor response profiles would have diverged a great deal over the course of tens of millions of years. We did not know a priori whether we would be able to correlate D. pseudoobscura ORNs with D. melanogaster counterparts.

**Figure 7:**
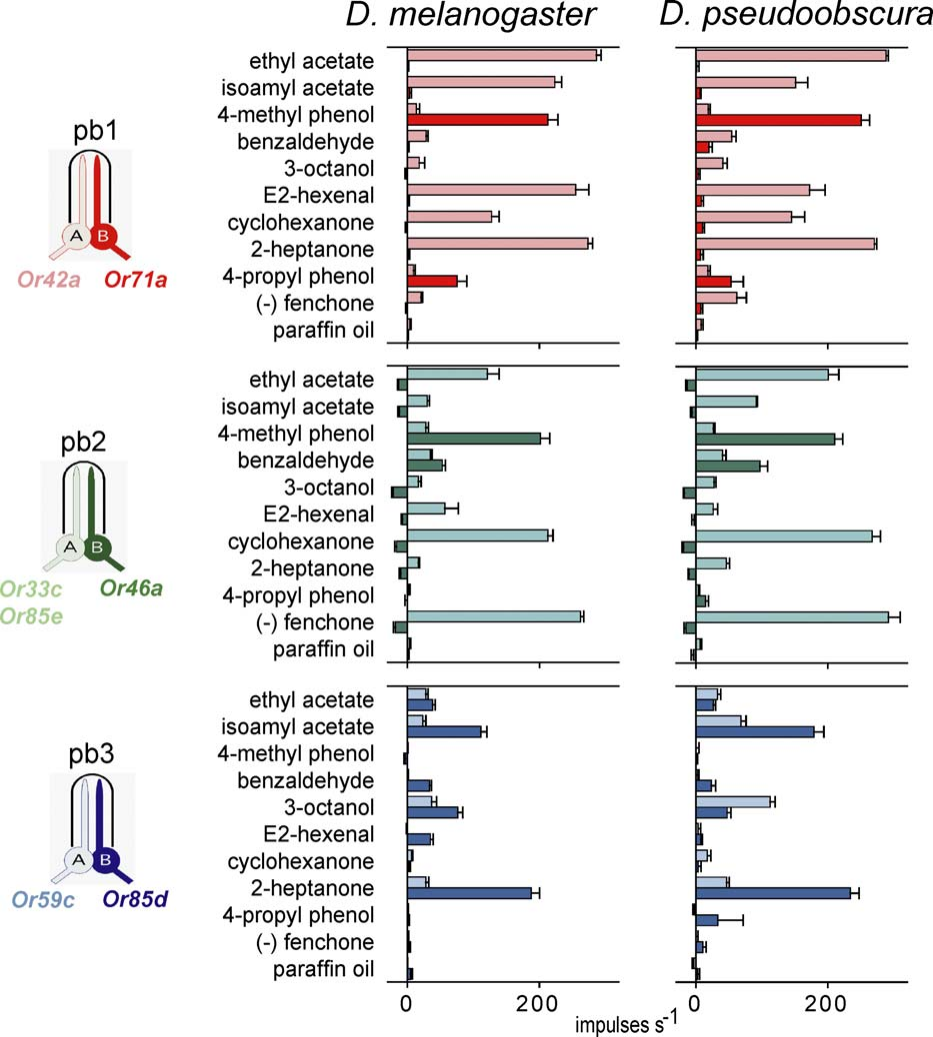
The Expression Program Is Conserved and Enhances ORN Sensitivity Electrophysiological analysis of the maxillary palp neurons from D. melanogaster and D. pseudoobscura. On the left is a receptor-to-neuron map of the D. melanogaster maxillary palp. The bar graphs on the right represent the responses in spikes per second, of different neurons, to a diagnostic set of odors that can be used to distinguish the functional expression of the different *Or* genes. Error bars = SEM; *n* = 10. For each sensillum type, the light-colored bar represents the A cell; the darker bar represents the B cell. The responses are measured as the change in action potential frequency following the onset of odorant stimulation. The map and the data for D. melanogaster are from [21]. We note that the maxillary palps of both species contain a small number of neurons that have not been well characterized yet.

We were surprised to find that the profiles of the maxillary palp ORNs are remarkably well conserved between these two species (Figure 7). Despite the tens of millions of years of separation, each ORN class in D. melanogaster has a counterpart in D. pseudoobscura, and their responses to a panel of ten diverse odorants are strikingly similar. Not only are the magnitudes of the responses well conserved, but the modes of the responses, i.e., excitation versus inhibition, are conserved. For example, both the pb2B ORN of *D. melanogaster* and its D. pseudoobscura counterpart are excited by 4-methyl phenol and inhibited by 3-octanol. The orthologous receptors show amino acid identity as low as 59% in the case of Or71a (Figure S3), and in no case exceeded 84%, the identity determined for Or42a. Thus pb1B in D. melanogaster, which expresses Or71a, shows the same specificity for 4-methyl phenol and 4-propyl phenol as the corresponding ORN in D. pseudoobscura, although Or71a is only 59% identical between the two species.

The conservation of odor response spectra allows us to determine that the stereotyped pairing of ORNs is also conserved in the two species. These results suggest that not only are the response spectra of the odor receptors conserved with respect to a diverse panel of odorants, but that the program of receptor gene expression is also conserved between these distantly related species.

### Search for Sensillum-Specific Elements

Given the success in identifying gene-specific elements required for the expression of individual *Or* genes in individual classes of ORNs, we asked whether the same approach could be used to identify sensillum-specific elements required uniquely by the *Or* genes that are expressed in the neighboring ORNs of a common sensillum. We searched for sensillum-specific elements conserved in the upstream regions of D. melanogaster and *D. pseudoobscura Or* genes. Only one element, AAATCAATTA, was found upstream of all orthologs expressed in a particular sensillum type (Figure S4A and [23]). Mutational analysis of this element in the *Or42a* promoter did not, however, appear to affect expression (Figures S4B–S4E). Furthermore, expression was not affected by mutation of the more proximal of the two copies of this element in the *Or71a* upstream region (unpublished data). These results suggest that this element is not required for expression in the pb1 sensillum.

## Discussion

We have analyzed the problem of how individual ORNs select which receptor genes to express, a fundamental problem that underlies all odor coding. In *Drosophila*, the foundation of olfactory perception is a stereotyped receptor-to-neuron map. The developmental process by which this map is constructed has been examined here using an analysis of evolutionary conservation as a point of departure.

We identified conserved, gene-specific elements flanking five maxillary palp receptor genes. Functional analysis of the six most highly conserved elements confirmed that elements upstream of four of these genes act either positively or negatively in gene regulation, thereby validating the experimental approach. Two elements did not appear to be required for normal gene regulation; however, it is possible that they act in a redundant fashion or that they mediate a response to such epigenetic factors as feeding status, mating status, or circadian rhythm, which we did not examine.

The elements varied in length from 7 to 19 bp; some of the longer ones could be composite sites that bind more than one factor. Several of the sites contain AT-rich cores, reminiscent of binding sites for certain classes of transcription factors including POU domain proteins. One element, 42a4, contains two iterations of an octamer, in an inverted repeat. Two elements, 46a1 and 71a3, overlap with a Dyad-1 element, CTA(N)_9_TAA, a positive regulatory element that is required for normal maxillary palp expression and that is found upstream of all of these maxillary palp *Or* genes [23]. The close juxtaposition of regulatory elements suggests an interaction among the regulatory proteins that they bind.

Our strategy for identifying these elements required that each be specific to a single maxillary palp *Or* gene. The identification of these elements reveals that each gene contains at least one unique element that is not shared with any other maxillary palp *Or* genes. This need not have been the case: the system could alternatively have been composed entirely of nonunique regulatory elements, each shared by multiple genes, but in unique combinations. In any case, in the maxillary palp the combinatorial code of *cis*-acting elements appears to include both unique and shared elements (e.g., Dyad-1).

**Figure 8:**
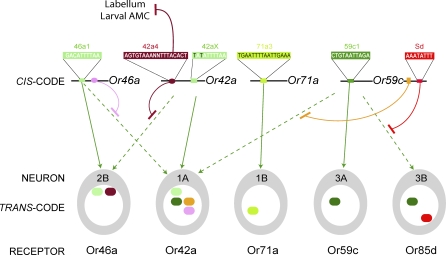
Model for Combinatorial Coding of Odor Receptor Gene Choice Conserved gene-specific regulatory elements, the genes that contain them, and the maxillary palp ORNs in which the genes are expressed, are depicted. Green elements are positive regulatory elements; red, pink, and orange elements are negative. Solid green arrows connect genes to the ORNs in which they are expressed in wild-type. These arrows originate from regulatory elements in cases where the elements have been shown to act positively, but do not imply that such elements alone are capable of directing proper expression. By contrast, 42ax has not been tested functionally and its arrow does not originate from this element. Dashed green arrows indicate ectopic expression driven by the indicated elements in the context of a minimal promoter, expression that in wild-type is repressed through the agency of other indicated elements, as represented by the curved red, pink, and orange lines. The pink and orange elements and their positions in *Or46a* and *Or59c*, respectively, have not been defined. The prefix “pb” has been deleted from the ORN designations. In the ORNs are ovals representing predicted transcription factors, color-coded according to the elements upon which they are presumed to act. For simplicity, a single factor (dark green; left column, second row), expressed in pb1A, pb3A, and pb3B, is proposed to act on 59c1, and a single factor (light green; left column, top row) expressed in both pb2B and pb1A is proposed to act on 46a1; more complicated models in which these elements are acted upon by multiple factors are also possible. Promoters are not drawn to scale. AMC, antenno-maxillary complex, which contains the dorsal organs.

The regulatory elements and the logic by which they operate are summarized in Figure 8. Positive regulatory elements direct expression in subsets of maxillary palp ORNs. Negative regulatory elements restrict this expression to a single ORN class. Overall, the correct expression pattern is determined by the interplay of positive and negative elements.

The negative regulation we have observed is highly specific. When the 42a4 element was ablated, *Or42a* misexpression was observed specifically in pb2B. One possible interpretation is that pb2B and pb1A, the cell that normally expresses *Or42a*, share a positively acting transcription factor that other ORNs lack. Thus the two ORNs with contexts that are permissive for *Or42a* expression are not neighboring ORNs that share a sensillum, but ORNs in different sensilla, with very different odor response profiles. Reciprocally, a positively acting element upstream of *Or46a*, which is expressed in pb2B, drives expression not only in pb2B but also in pb1A. This connection between pb1A and pb2B suggests a developmental relationship that remains to be defined in mechanistic terms.

This study has concentrated on receptor gene choice in the maxillary palp, on account of its numerical simplicity. Does a system of molecular zip codes also underlie the process of receptor gene choice across the entire odor receptor repertoire? In addition to the seven maxillary palp receptors, the *Or* gene family contains 53 other members expressed in the antenna or the larval olfactory system [19,30–32]. Using a comparative bioinformatic approach, we performed a large-scale analysis of sequence conservation in the 500 bp upstream of each of 42 *Or* genes across all 12 *Drosophila* species (Figure S5 and Text S1).**** We found great diversity in the number, lengths, and distribution of highly conserved upstream regions. Within the most highly conserved of these regions we identified a variety of elements that are shared among subsets of *Or* genes (Figure S6A and S6B). This analysis, then, reveals a combinatorial structure to the organization of shared elements upstream of these receptor genes. This pattern supports a model in which a combinatorial code of positive and negative regulatory elements dictates the proper expression of each *Or* gene.

What kind of proteins accomplish this regulation? In C. elegans, several kinds of transcription factors have been elegantly shown to play roles in specifying ORN identity and receptor expression [33]. In the mouse, a LIM-homeodomain protein, Lhx2, is required for normal ORN differentiation and expression of OR genes [34,35]. In *Drosophila* the POU domain protein Acj6 is required for the expression of a subset of *Or* genes [36]. We have also shown that Sd, a TEA domain-containing transcription factor, is critical in restricting the expression of some *Or* genes to their proper ORNs. Sd has been shown to act as a repressor in other systems and in fact is required for normal taste behavior in both larvae and adults [37]. Another aspect of receptor gene choice depends on proteins of the Notch pathway: receptor choice in neighboring ORNs of a sensillum appears to be coordinated via asymmetric segregation of regulatory factors from a common progenitor [23,38].

Some elements that are essential to odor receptor gene choice are also located upstream of genes required for axon guidance and sorting (Figure S7 and Text S1). The presence and positions of these elements have been conserved for tens of millions of years of evolution. The presence of *Or* regulatory elements upstream of ORN axon-guidance genes could reflect a relationship between receptor gene choice and axon targeting. In addition to selecting particular *Or* genes for expression, ORNs send axons to particular glomeruli in the antennal lobe of the brain. ORNs that express the same *Or* gene send axons to the same glomerulus [16,19]. Thus the olfactory system contains both a stereotyped receptor-to-neuron map and a stereotyped connectivity map in the antennal lobes. The tight coordination between receptor gene choice and axonal projection could in principle arise in part from overlap in the mechanisms underlying these processes. In mammals, odor receptors play a role in ORN targeting [39–41]. In *Drosophila*, ORN targeting does not require the receptors [20], but could require the regulatory apparatus used to express the receptors. Acj6 provides an example of a link between the two processes: it acts both in receptor expression and ORN axon targeting (Figure 2) [13,25]. Moreover, we have found that Acj6 is required for the activity of one of the regulatory elements identified here.

We found a remarkable similarity of function between the maxillary palp ORNs of two species that diverged more than tens of millions of years ago. We had expected that over this time interval, the odor specificities of the ORNs would have diverged markedly to serve differing needs of the two evolving species. Instead, every ORN class showed strikingly similar responses, with few exceptions. The results show that two odor receptors can differ a great deal in amino acid sequence and still exhibit a very similar odor specificity.

The organization of the organ in the two species is also identical, in that corresponding ORNs are combined according to the same pairing rules. This high degree of conservation suggests a critical role for the maxillary palp in odor coding and in the generation of olfactory-driven behavior. The conservation of regulatory elements and organization also suggests that the two species use common mechanisms to specify the receptor-to-neuron map.

The regulatory challenge confronted by the *Drosophila* olfactory system represents an extreme among problems of gene regulation. It requires the storage and deployment of a great deal of information. Our data support a model in which *Or* gene expression is controlled by a system of molecular zip codes. Each *Or* gene contains elements that dictate expression in the proper olfactory organ [23], positive regulatory elements that specify expression in a subset of ORN classes, and negative regulatory elements that restrict expression to a single ORN class. This logic and the components that execute it have solved such a challenging problem with such efficiency that they have apparently been well conserved for tens of millions of years.

## Materials and Methods

### *Drosophila* stocks, genetics, and transformation.

*Drosophila* stocks were raised at 25 °C. Wild-type flies were Canton-S unless otherwise indicated. *sd^1^* and *sd^ETX4^*, referred to here as *sd*{*PlacZ*}, were obtained from the *Drosophila* Stock Center (Bloomington, Indiana). *sd^SG29.1^* was a gift from S. Cohen. *D. pseudoobscura* was from the *Drosophila* Species Resource Center (Tucson, Arizona). *w; UAS-mCD8-GFP/CyO;UAS-mCD8-GFP* was used as a source of GFP unless otherwise indicated. All DNA constructs were sequenced and then injected along with Δ2,3 transposase plasmid into *w^1118^* flies. Multiple transgenic lines, in most cases eight, were generated and tested for each construct.

### Bioinformatics.

To identify gene-specific conserved sequences in the upstream maxillary palp *Or* genes, we used ClustalW alignments and DOT-PLOT analysis (MacVector ). To map identified *cis-*elements to sequences and identify overrepresented motifs, the DNA-PATTERN (STRINGS) and OLIGO-ANALYSIS programs were used at the RSA tools website (http://rsat.scmbb.ulb.ac.be/rsat/). For the identification of conserved sequences the *Drosophila* genome browser at http://genome.ucsc.edu/ was used. A multiple alignment was constructed using MULTIZ from the best-in-genome pairwise alignments generated by BLASTZ. Large-scale predictions of conserved elements were obtained from the multiple alignments using the PhastCons program with the most-conserved option. Shared elements were identified using the OLIGO-ANALYSIS program at the RSA tools website.

### Mutant promoter constructs.

The wild-type *Or42a* 4.1-kb promoter-*GAL4* construct has been described previously [21]. In (42a4)-*GAL4*, the 42a4 element, which contains an inverted repeat (AGTGTAAANNTTTACACT), was mutated to (AGTG–––TTT*GGATCC*), resulting in a deletion within the first half-element and the substitution of a *BamHI* recognition sequence in the second half-element (italicized). This was accomplished by PCR amplification of two promoter fragments, one terminating immediately upstream of the TAAA in the first octamer of the 42a4 element, and the second wasa fragment extending from immediately downstream of this element to the start codon of *Or42a*. Primers for these PCR reactions contained the *BamHI* restriction site in place of ACACTT. The PCR products were AT-cloned into pGEM-T Easy. Subsequent ligation of the two PCR fragments resulted in the desired replacement in the context of the *Or42a* 4.1-kb promoter-transgene.

In the (42a5+6)-*GAL4* construct a small deletion was designed to delete both the 42a5 and the 42a6 elements, which are separated by 3 bp (TGTGAACGATTGCAGCCTG). This was achieved by using a similar approach as for 42a(4)-*GAL4*, but in this case the two primers, containing *BamHI* sites at their ends, were designed to start immediately upstream of 42a6 and immediately downstream of 42a5. Ligation of the appropriate fragments led to the replacement of the entire 19-bp region, comprising the two elements, by a *BamHI* site.

In the (46a1)-*GAL4* construct the 46a1 element (GACATTTTAA) was mutated by replacing the first six bases with a *BamHI* restriction site. This was achieved using a PCR cloning strategy similar to the ones described for the *Or42a* constructs.

In the (71a3)-*GAL4* construct the 71a3 element (TGAATTTTAATTGAAA) was mutated to (GCTAGCTTAATTGAAA) by replacing the first six bases with a *NheI* restriction site using a PCR cloning strategy similar to the one described earlier, resulting in the desired mutation in the context of the *Or71a* 5′ + 3′-*GAL4* construct.

We note that 46a1 and 71a3 each overlaps with a Dyad-1 motif, CTA(N)_9_TAA, a positive regulatory element that is required for expression of *Or* genes in the maxillary palp [23]; the mutations of 46a1 and 71a3 were designed so as not to affect the Dyad-1 motif.

The *Or59c* 2.1-kb promoter-*GAL4* construct has been described in [21] and has been shown to express in a large number of non-endogenous cells in the palp. The *Or59c* 5′ + 3′-*GAL4* was constructed by cloning a 0.5-kb fragment of DNA that lies immediately downstream of the *Or59c* stop codon into the *SpeI/BamHI* site that is positioned downstream of the *GAL4*-hsp70 3′ UTR in pG4PN. The 0.5-kb fragment was PCR-amplified from Canton-S genomic DNA with primers designed to add a *Spe1* site to the 5′ end and a *BamHI* site to the 3′end.

The *Or59c* (5′ + 3′ direct)-*GAL4* construct was made in several steps. First the 0.5-kb fragment of DNA immediately downstream of the *Or59c* stop codon, described above, was cloned into the *BamHI/Spe1* site of pSK+ to generate plasmid pSK3'. Second, the *GAL4* coding region was cloned as a *HindIII* fragment into pSK3' to yield pSKGAL4. The *Or59c* 5′ region was excised from the *Or59c* 2.1kb-*GAL4* vector using *KpnI/NotI*(blunted) and it was *KpnI*/blunt cloned into the *Kpn1/Apa1*(blunted) site of the pSKGAL4 plasmid. Finally the *KpnI*/*SpeI* fragment from this plasmid was ligated with the *KpnI/SpeI* fragment of pG4PN to yield *Or59c* complex-*GAL4.*

### Minimal promoter constructs.

Complementary pairs of oligonucleotides were designed such that upon annealing, they would yield a double-stranded DNA fragment that includes multiple copies of the corresponding conserved elements and overhangs on either side for EcoR1 restriction enzyme sites. These fragments were cloned directly into the EcoR1 site of the *pPTGAL Drosophila* transformation vector [42]. The 46a1 sequence was GACATTTTAAATGCC*CTA*ATGACATTT*TAA*ATGCC*CTA*ATGACATTT*TAA*ATGCC*CTA*ATGACATTT*TAA* *.* The 71a3 sequence was *CTA*ATTGAATTT*TAA*TTGAAACGTCA*CTA*ATTGAATTT*TAA*TTGAAACGTCA*CTA*ATTGAATTT*TAA*TTGAAACGTCA. The 59c1 sequence was GCAAACTGTAATTAGAGGACCGCAAACTGTAATTAGAGGACCGCAAACTGTAATTAGAGGACCGCAAACTGTAATTAGAGGACC. We note that the constructs for 46a1 and 71a3 contain Dyad-1 motifs, but these motifs are not sufficient to drive expression in the maxillary palp [23]. The underlined sequences indicate the gene-specific elements, and the italicized sequences indicate the Dyad-1 sequences. For each minimal promoter construct, at least two independent lines were examined, and *n* > 20 maxillary palps were examined for each line.

### In situ hybridization and immunolabeling.

In situ hybridization and immunohistochemical localization were performed as in [21]. Mouse anti-βGAL antibody (1:1000), and rabbit anti-GFP (1:250) were obtained from Promega.

To generate a high-resolution map of the nuclei of the three sensilla types (Figure 1C), (*42a4*)*-GAL4/UAS-GFP*; *UAS-GFP/+* was used to label pb1A and pb2B in green, and *Or46a* and *Or59c* in situ hybridization probes were used to label with red the pb2B and pb3A cells, respectively. Thus pb1A was labeled green, pb2B was labeled yellow (red and green); pb3A was labeled red. Confocal Z-stacks consisting of nine optical sections of each palp were analyzed in Photoshop. Positions of the labeled nuclei were manually marked with the corresponding color at each optical plane, and the 9 stacks were compressed to generate a 2-D representation of all the labeled neurons.

### Electrophysiology.

Odors were delivered and action potentials were recorded as described previously [20] and in Text S1.

## Supporting Information

Figure S1 Conservation of Gene-Specific Elements across 12 Species (A) DOT-PLOT graphs of the 500-bp region upstream of indicated genes from two species. All diagonals 7 bp or greater are indicated.
(B) Pairwise alignment of each species to the 500-bp upstream of each indicated D. melanogaster gene (400 bp for *Or46a*, up to the adjacent gene), generated from the UCSC genome browser. Grayscale density plots indicate conservation. Arrow indicates the position of ATG translation start site and direction of translation. Colored boxes indicate positions of the best-conserved motifs. Double lines in alignment indicate unalignable gaps. In the scale at the bottom of the panel, each tick represents 100 bp. On the right are the sequence alignments for the best-conserved gene-specific motifs for each *Or* gene.
(C) Sequence alignments of the other gene-specific conserved motifs. The D. melanogaster 46a2 element is not shown because it occurs at a different position from those of D. pseudoobscura and *D. persimilis.*
(469 KB PDF)

Figure S2 Misexpression of *Or85d* 5′ Promoter Construct (A) Conservation of the Sd binding site at *Or59c*.
(B) Optical sections from *Or85d 5′-GAL4/UAS-GFP;UAS-GFP/+* maxillary palps labeled with an anti-GFP antibody and the indicated RNA probes.
(212 KB PDF)

Figure S3 Orthologs of *Or71a*
Amino acid sequence alignments of *Or71a* genes from D. melanogaster and D. pseudoobscura. Red bars indicate positions of predicted transmembrane regions.
(54 KB PDF)

Figure S4 Functional Analysis of a Candidate Sensillum-Specific Element (A) Map of the candidate sensillum-specific conserved motifs in the upstream sequences of the maxillary palp *Or* genes. Positions and sequences of three partially conserved and one completely conserved element are indicated.
Expression of GFP driven by the wild-type promoter construct (B), and a mutant construct in which the candidate pb1 element is abolished (C). Images are Z-compressions of confocal stacks.
(D) Optical micrograph(s) of *42a pb1*)*-GAL4/UAS-GFP;UAS-GFP/+* maxillary palps labeled with anti-GFP antibody and an Or71a RNA in situ hybridization probe. Arrowheads indicate expression in appropriate neighboring paired cells. Flies contained one copy of the *Or-GAL4* constructs and two copies of *UAS-mCD8::GFP*.
(E) Numbers of GFP^+^ cells in maxillary palps containing wild-type and mutant Or42a-GAL4 constructs. *n* = 9 maxillary palps.
(350 KB PDF)

Figure S5 Conserved Sequences Upstream of Antennal and Larval *Or* Genes (A) Pairwise alignment of the 500-bp upstream region of each indicated *Or* gene of each species to the corresponding D. melanogaster sequence, generated from the UCSC genome browser. For the identification of conserved regions upstream of the 42 antennal and larval *Or* genes, we used a phylogenetic hidden-Markov model program, PhastCons, to automate the procedure [2,3,16]. The 11 species compared to D. melanogaster are in the same order as in Figure 1D. Conservation scores are displayed as a “wiggle” histogram where height reflects the magnitude of the score. Grayscale density plots underneath indicate conservation. Double lines in alignment indicate an unalignable sequence, single lines indicate absence of sequence. “+” indicates transcription of a gene is from left to right; “–” indicates right to left. The dark boxes underneath the density plots indicate the positions of the conserved sequences identified by the PhastCons program. The best conserved sequence for each gene is indicated in Figure S6a.
The *Or* genes showed remarkable variation in the number, lengths, and distribution of these conserved DNA sequences. The number of sequences ranged from 13, in the case of *Or45b*, to none, in the cases of *Or13a*, *Or22b*, *Or43b*, and *Or85a*, with a mean number of 3.7 elements/gene. The lengths of individual sequences identified by this procedure ranged from 186 bp to 9 bp. In some cases, the conserved sequences were primarily located in a single block, as in *Or35a* and *Or69a*, either near (*Or35a*) or far (*Or69a*) from the translation start site; in other cases the conserved sequences were distributed more evenly across the entire 500-bp region, as in the case of *Or56a*. We did not find that highly conserved receptors contain more highly conserved upstream regions than poorly conserved receptors (unpublished data).
(2.52 MB PDF)

Figure S6 A Combinatorial Code of Elements within Conserved Regions (A) Sequences of the highest scoring PhastCons conserved region for each gene, and elements shared among the best conserved PhastCons sequences for each gene. Colored boxes indicate the presence of an element in the indicated PhastCon region of each D. melanogaster gene. The sensillum type in which each gene is expressed is indicated: ab, antennal basiconic; ac, antennal coeloconic; ai, antennal intermediate; L, larval olfactory organ (from [5–8,19]). In some cases, the indicated PhastCon sequence contains the reverse complement of the indicated element. Also indicated are the presence of predicted binding sites for transcription factors Lz and Sd in the 500 bp of sequence upstream of each receptor. Each indicated Lz and Sd binding site is conserved in at least five or more *Drosophila* species, with greater than 90% of residues identical within the conserved sequences. a, CAATTA; b, TAATTA; c, AATTAT; d, AATTAC; e, ATTACA; f, GCAAATT; g, TTGCATA; h, GCTCATTA; Lz, RACCRCA; Sd, AAATATTT.
(B) Expected and observed occurrences of each element.
(128 KB PDF)

Figure S7 Conservation of Elements Upstream of Axon-Guidance Genes (A) Map of elements in the upstream sequences of axon-guidance genes (table adapted from [1]). The dots surrounding the 42a4 symbol indicate that a single iteration of the AGTGTAAA sequence is observed.
(B) Sequence alignment of elements upstream of axon-guidance genes. The Oligo-1 element upstream of *Ptp10D* is not shown because although present in 5 species, its position is not well-conserved.
(91 KB PDF)

Text S1 Shared *cis*-Elements in Antennal and Larval *Or G*enes and Axon-Guidance Molecules (51 KB DOC)

## Abbreviations

GFP: green fluorescent protein
ORN: olfactory receptor neuron
UTR: untranslated region
