# Supplementary material for: A Regulatory Code for Neuron-Specific Odor Receptor Expression
Source: PLoS Biol. 2008 May 27;6(5):e125. doi: 10.1371/journal.pbio.0060125 (PMC2430909; doi:10.1371/journal.pbio.0060125)
Supplement: Figure S1 — (A) DOT-PLOT graphs of the 500-bp region upstream of indicated genes from two species. All diagonals 7 bp or greater are indicated. (B) Pairwise alignment of each species to the 500-bp upstream of each indicated D. melanogaster gene (400 bp for Or46a, up to the adjacent gene), generated from the UCSC genome browser. Grayscale density plots indicate conservation. Arrow indicates the position of ATG translation start site and direction of translation. Colored boxes indicate positions of the best-conserved motifs. Double lines in alignment indicate unalignable gaps. In the scale at the bottom of the panel, each tick represents 100 bp. On the right are the sequence alignments for the best-conserved gene-specific motifs for each Or gene. (C) Sequence alignments of the other gene-specific conserved motifs. The D. melanogaster 46a2 element is not shown because it occurs at a different position from those of D. pseudoobscura and D. persimilis. (469 KB PDF) [file pbio.0060125.sg001.pdf]

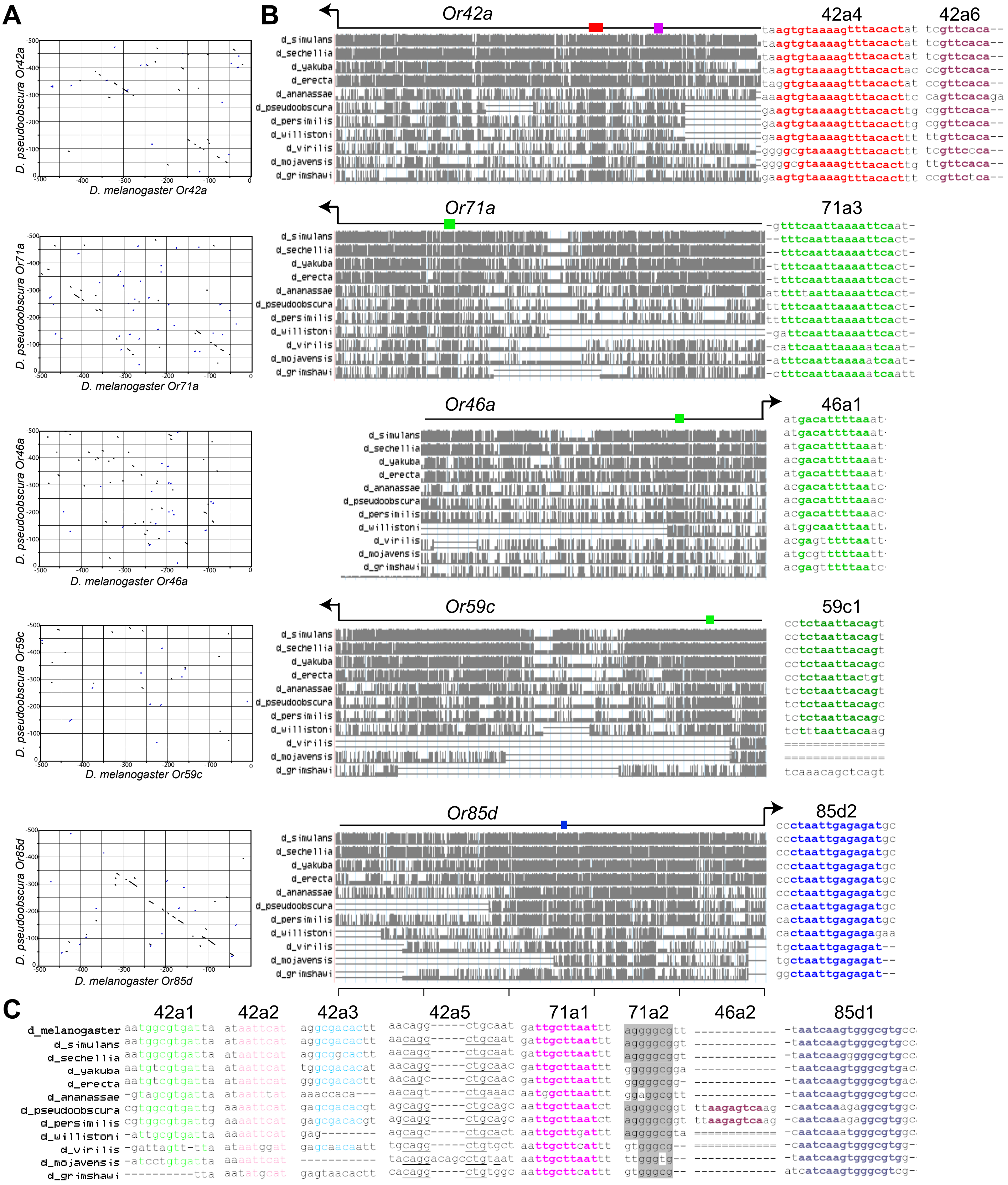
A
D. pseudoobscura Or42a
D. melanogaster Or42a
D. pseudoobscura Or71a
D. melanogaster Or71a
D. pseudoobscura Or46a
D. melanogaster Or46a
D. pseudoobscura Or59c
D. melanogaster Or59c
D. pseudoobscura Or85d
D. melanogaster Or85d
B
Or42a
42a4
42a6
d_simulans taagtgtaaaagtttacactat tcgttcaca--
d_sechellia taagtgtaaaagtttacactat tcgttcaca--
d_yakuba taagtgtaaaagtttacactac ccgttcaca--
d_erecta taggtgtaaaagtttacactat ccgttcaca--
d_ananassae aaagtgtaaaagtttacacttc cagttcacaga
d_pseudoobscura gaagtgtaaaagtttacacttg cggttcaca--
d_persimilis gaagtgtaaaagtttacacttg cggttcaca--
d_willistoni gaagtgtaaaagtttacacttt ttgttcaca--
d_virilis ggggcgtaaaagtttacacttt tcgttccca--
d_mojavensis ggggcgtaaaagtttacacttg ttgttcaca--
d_grimshawi gaagtgtaaaagtttacacttt ccgttctca--
Or71a
71a3
d_simulans -gtttcaattaaaattcaat-
d_sechellia --tttcaattaaaattcact-
d_yakuba --tttcaattaaaattcact-
d_erecta -ttttcaattaaaattcact-
d_ananassae -ttttcaattaaaattcact-
d_pseudoobscura attttaattaaaattcact-
d_persimilis tttttcaattaaaattcact-
d_willistoni tttttcaattaaaattcact-
d_virilis -gattcaattaaaattcact-
d_mojavensis -cattcaattaaaaatcaat-
d_grimshawi -atttcaattaaaaatcaat-
-ctttcaattaaaaatcaatt
Or46a
46a1
d_simulans atgacattttaaat
d_sechellia atgacattttaaat
d_yakuba atgacattttaaat
d_erecta acgacattttaaat
d_ananassae atgacattttaaat
d_pseudoobscura acgacattttaatc
d_persimilis acgacattttaaac
d_willistoni acgacattttaaac
d_virilis atggcaatttaatt
d_mojavensis acgagttttaatt
d_grimshawi atgcgttttaatt
acgagttttaatc
Or59c
59c1
d_simulans cctctaattacagt
d_sechellia cctctaattacagt
d_yakuba cctctaattacagt
d_erecta cctctaattacagc
d_ananassae cctctaattactgt
d_pseudoobscura tctctaattacagt
d_persimilis tctctaattacagc
d_willistoni tctctaattacagc
d_virilis tcttaattacaag
d_mojavensis ==============
d_grimshawi ==============
tcaaacagctcagt
Or85d
85d2
d_simulans ccctaattgagagatgc
d_sechellia ccctaattgagagatgc
d_yakuba ccctaattgagagatgc
d_erecta ccctaattgagagatgc
d_ananassae ccctaattgagagatgc
d_pseudoobscura ccctaattgagagatgc
d_persimilis cactaattgagagatgc
d_willistoni cactaattgagagatgc
d_virilis cactaattgagagagaa
d_mojavensis tgctaattgagagat--
d_grimshawi tgctaattgagagat--
ggctaattgagagat--
C
42a1 42a2 42a3 42a5 71a1 71a2 46a2 85d1
d_melanogaster aatggcgtgatta ataattcat aggcgacactt aacagg-----ctgcaat gattgcttaattt aggggcgtt ------------ -taatcaagtgggcgtgcc
d_simulans aatggcgtgatta ataattcat aggcgacactt aacagg-----ctgcaat gattgcttaattt agggggcggt ------------ -taatcaagtgggcgtgcc
d_sechellia aatggcgtgatta ataattcat aggcggcactt aacagg-----ctgcaat gattgcttaattt aggggcggt ------------ -taatcaaggggcgtgcc
d_yakuba aatgtcgtgatta ataattcat tggcgacacat aacagg-----ctgcaac gattgcttaattt ggggggcgga ------------ -taatcaagtgggcgtgac
d_erecta aatgtcgtgatta ataattcat gggcgacactt aacagc-----ctgcaac gattgcttaattt gggggcgg- ------------ -taatcaagtgggcgtgac
d_ananassae -gtagcgtgatta ataatttat aaaccaca--- aacagt-----ctgaaac aatggcttaattt ggaggcgtt ------------ -taatcaagtgggcgtgcc
d_pseudoobscura cgtggcgtgattg aaaattcat gagcgacacgt agcagg-----ctgcagc aattgcttaatct aggggcggt ttaagagtcaag -caatcaaagaggcgtggc
d_persimilis cgtggcgtgattg aaaattcat gagcgacacgt agcagg-----ctgcagc aattgcttaatct aggggcggt ttaagagtcaag -caatcaaagaggcgtggc
d_willistoni -attgcgtgatta aaaattcat gag-------- agcagc-----ctgcact aattgcttgattt aggggcgta ============ -caatcaaatgggcgtggc
d_virilis -gattagtt-tta ataatggat gagcaacaatt tgcagg-----ctgtgct aattgcttcattt gtgggcg-- ============ -caatcaagtgggcgtgg-
d_mojavensis -atcctgtgatta aaaattcat ----------- tacaggacagcctgtaat aattgcttaatct ttgggtg-- ------------ -taatcaagtgggcgtgg-
d_grimshawi ----------tta aatatgcat gagtaacactt cacagg-----ctgtggc aattgcttcattt gtgggcg-- ------------ atcatcaagtgggcgtcg-
