# Supplementary material for: A Regulatory Code for Neuron-Specific Odor Receptor Expression
Source: PLoS Biol. 2008 May 27;6(5):e125. doi: 10.1371/journal.pbio.0060125 (PMC2430909; doi:10.1371/journal.pbio.0060125)

A

```
D. melanogaster  gcaaatatttgg
    D. simulans  gcaaatattggg
   D. sechellia  gcaaatattggg
      D. yakuba  gcaaatattagg
      D. erecta  gcaaatattggg
   D. ananassae  ============
D. pseudoobscura  gaaaatattg--
   D. persimilis  gaaaatattgaa
    D. willistoni  ============
      D. virilis  attaataattca
   D. mojavensis  gttaatataaaa
    D. grimshawi  tttaagatttgc
```

B

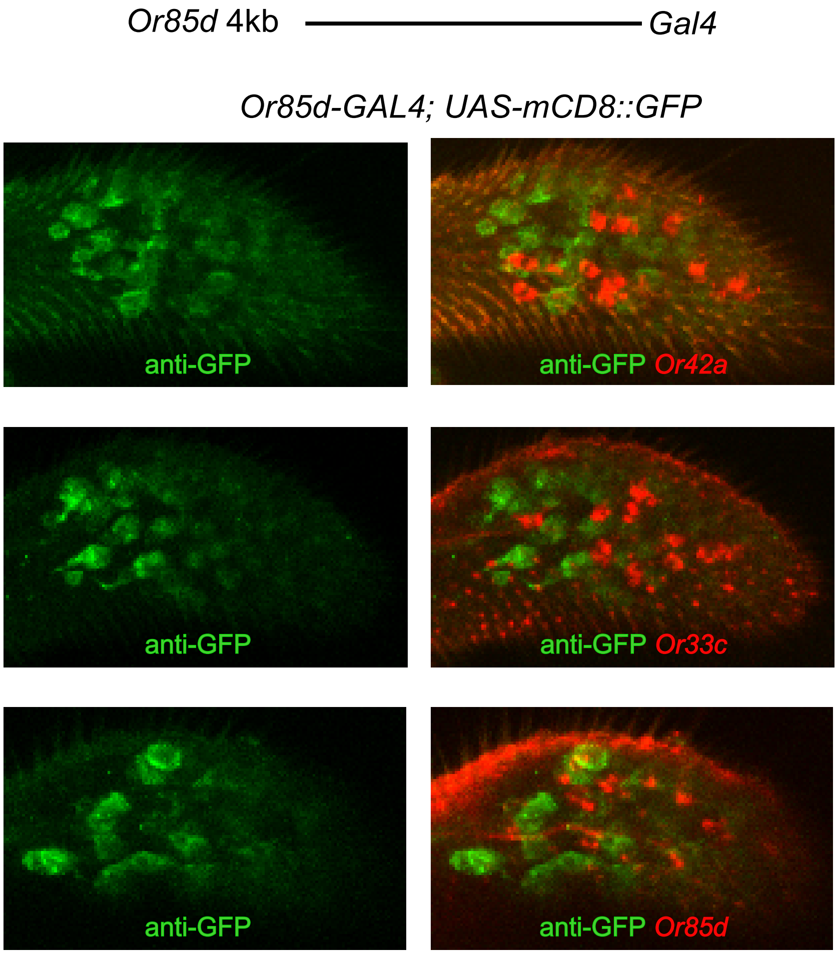

Supplement: Figure S2 — (A) Conservation of the Sd binding site at Or59c. (B) Optical sections from Or85d 5′-GAL4/UAS-GFP;UAS-GFP/+ maxillary palps labeled with an anti-GFP antibody and the indicated RNA probes. (212 KB PDF) [file pbio.0060125.sg002.pdf]
