# Supplementary material for: A Regulatory Code for Neuron-Specific Odor Receptor Expression
Source: PLoS Biol. 2008 May 27;6(5):e125. doi: 10.1371/journal.pbio.0060125 (PMC2430909; doi:10.1371/journal.pbio.0060125)
Supplement: Figure S3 — Amino acid sequence alignments of Or71a genes from D. melanogaster and D. pseudoobscura. Red bars indicate positions of predicted transmembrane regions. (54 KB PDF) [file pbio.0060125.sg003.pdf]

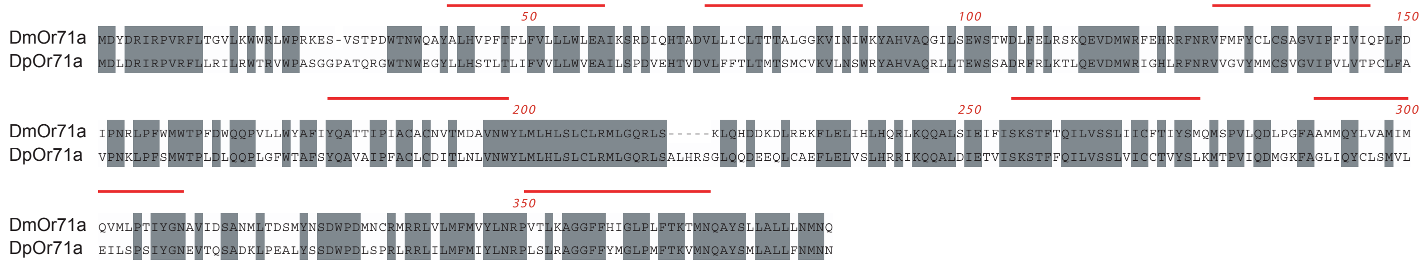
50
100
150
DmOr71a MDYDRIRPVRFLTGVLKWWRLWPRKES-VSTPDWTNWQAYALHVPFTFLFVLLLWLEAIKSRDIQHTADVLLICLTTTTALGGKVINIWKYAHVAQGILSEWSTWDLFELRSKQEVDMWRFEHRRFNRVFMFYCLCSAGVIPFIVIQPLFD
DpOr71a MDLDRIRPVRFLLRILRWTRVWPASGGPATQRGWTNWEGYLLHSTLTLIFVVLLWVEAILSPDVEHTVDVLFFTLTMTSMCVKVLNSWRYAHVAQRLLTEWSSADRFRLKTLQEVDMWRIGHLRFNRVVGVYMMCSVGVIPVLVTPCLFA
200
250
300
DmOr71a IPNRLPFWMWTPFDWQQPVLLWYAFIYQATTIPIACACNVTMDAVNWYLMLHLSLCLRMLGQRLS-----KLQHDDKDLREKFLELIHLHQRLKQQALSIEIFISKSTFTQILVSSLIICFTIYSMQMSPVLQDLPGFAAMMQYLVAMIM
DpOr71a VPNKLPFSMWTPLDLQQPLGFWTAFSYQAVAIPFACLCDITLNLVNWYLMLHLSLCLRMLGQRLSALHRSGLQQDEEQLCAEFLELVSLHRRIKQQALDIETVISKSTFFQILVSSLVICCTVYSLKMTPVIQDMGKFAGLIQYCLSMVL
350
DmOr71a QVMLPTIYGNAVIDSANMLTDSMYNSDWPDMNCRMRRLVLMFMVYLNRPVTLKAGGFFHIGLPLFTKTMNQAYSLLALLLNMNQ
DpOr71a EILSPSIYGNEVTQSADKLPEALYSSDWPDLSPRLRRLILMFMIYLNRPLSLRAGGFFYMGLPMFTKVMNQAYSMLALLFNMNN
