# Supplementary figures and images for: A Regulatory Code for Neuron-Specific Odor Receptor Expression
Source: PLoS Biol. 2008 May 27;6(5):e125. doi: 10.1371/journal.pbio.0060125 (PMC2430909; doi:10.1371/journal.pbio.0060125)

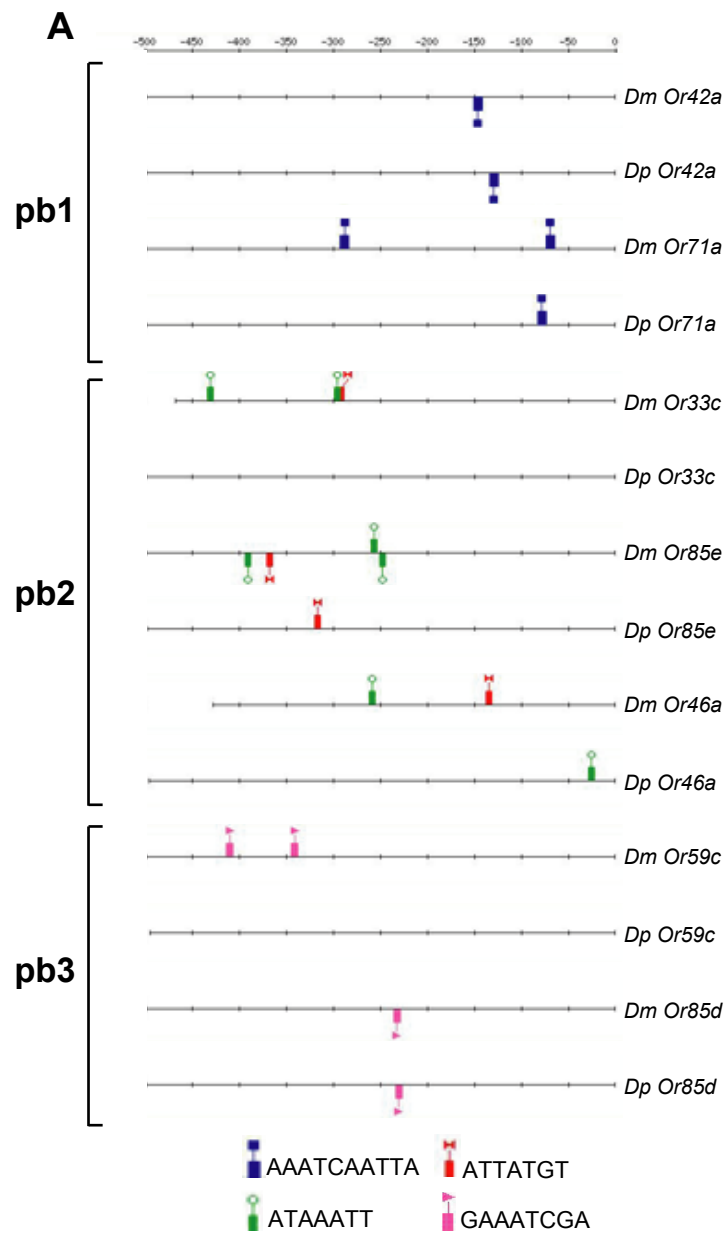

**B**

Or42a 4.1kb

AAATCAATTA

GAL4

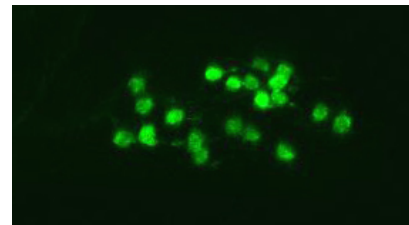

**C**

(42a pb1)

AAGCTAGCTA

GAL4

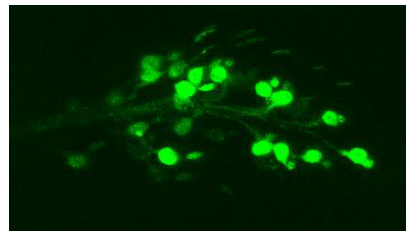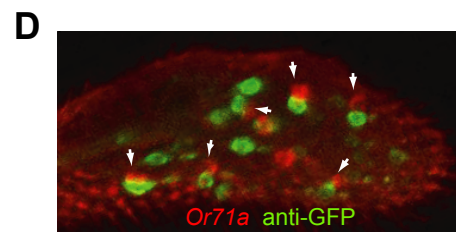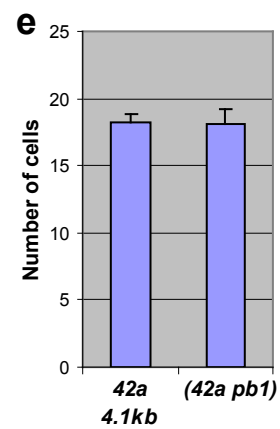

Supplement: Figure S4 — (A) Map of the candidate sensillum-specific conserved motifs in the upstream sequences of the maxillary palp Or genes. Positions and sequences of three partially conserved and one completely conserved element are indicated. Expression of GFP driven by the wild-type promoter construct (B), and a mutant construct in which the candidate pb1 element is abolished (C). Images are Z-compressions of confocal stacks. (D) Optical micrograph(s) of 42a pb1)-GAL4/UAS-GFP;UAS-GFP/+ maxillary palps labeled with anti-GFP antibody and an Or71a RNA in situ hybridization probe. Arrowheads indicate expression in appropriate neighboring paired cells. Flies contained one copy of the Or-GAL4 constructs and two copies of UAS-mCD8::GFP. (E) Numbers of GFP+ cells in maxillary palps containing wild-type and mutant Or42a-GAL4 constructs. n = 9 maxillary palps. (350 KB PDF) [file pbio.0060125.sg004.pdf]

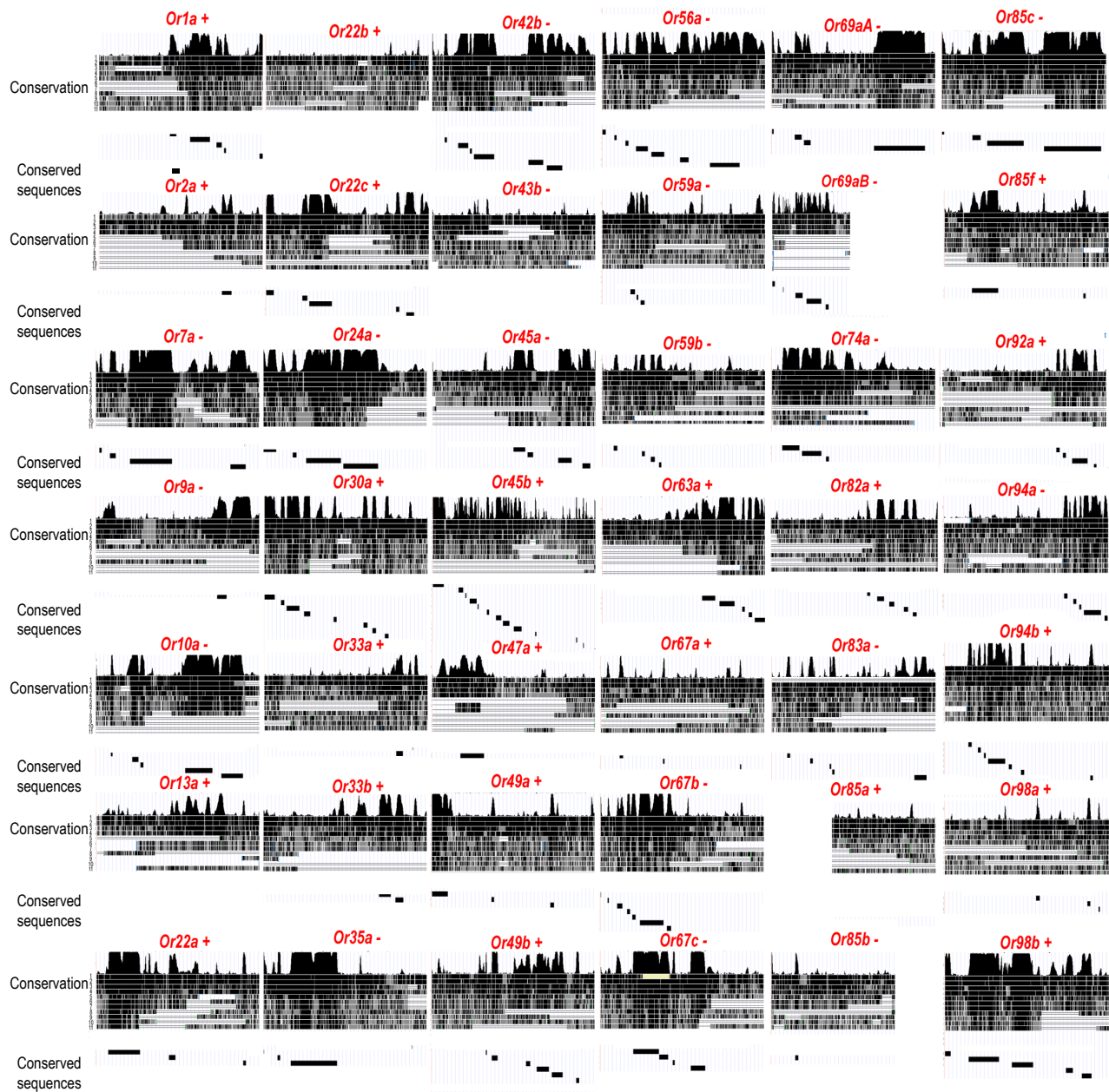

Supplement: Figure S5 — (A) Pairwise alignment of the 500-bp upstream region of each indicated Or gene of each species to the corresponding D. melanogaster sequence, generated from the UCSC genome browser. For the identification of conserved regions upstream of the 42 antennal and larval Or genes, we used a phylogenetic hidden-Markov model program, PhastCons, to automate the procedure [2,3,16]. The 11 species compared to D. melanogaster are in the same order as in Figure 1D. Conservation scores are displayed as a “wiggle” histogram where height reflects the magnitude of the score. Grayscale density plots underneath indicate conservation. Double lines in alignment indicate an unalignable sequence, single lines indicate absence of sequence. “+” indicates transcription of a gene is from left to right; “–” indicates right to left. The dark boxes underneath the density plots indicate the positions of the conserved sequences identified by the PhastCons program. The best conserved sequence for each gene is indicated in Figure S6a. The Or genes showed remarkable variation in the number, lengths, and distribution of these conserved DNA sequences. The number of sequences ranged from 13, in the case of Or45b, to none, in the cases of Or13a, Or22b, Or43b, and Or85a, with a mean number of 3.7 elements/gene. The lengths of individual sequences identified by this procedure ranged from 186 bp to 9 bp. In some cases, the conserved sequences were primarily located in a single block, as in Or35a and Or69a, either near (Or35a) or far (Or69a) from the translation start site; in other cases the conserved sequences were distributed more evenly across the entire 500-bp region, as in the case of Or56a. We did not find that highly conserved receptors contain more highly conserved upstream regions than poorly conserved receptors (unpublished data). (2.52 MB PDF) [file pbio.0060125.sg005.pdf]
