# Supplementary material for: A Regulatory Code for Neuron-Specific Odor Receptor Expression
Source: PLoS Biol. 2008 May 27;6(5):e125. doi: 10.1371/journal.pbio.0060125 (PMC2430909; doi:10.1371/journal.pbio.0060125)
Supplement: Figure S6 — (A) Sequences of the highest scoring PhastCons conserved region for each gene, and elements shared among the best conserved PhastCons sequences for each gene. Colored boxes indicate the presence of an element in the indicated PhastCon region of each D. melanogaster gene. The sensillum type in which each gene is expressed is indicated: ab, antennal basiconic; ac, antennal coeloconic; ai, antennal intermediate; L, larval olfactory organ (from [5–8,19]). In some cases, the indicated PhastCon sequence contains the reverse complement of the indicated element. Also indicated are the presence of predicted binding sites for transcription factors Lz and Sd in the 500 bp of sequence upstream of each receptor. Each indicated Lz and Sd binding site is conserved in at least five or more Drosophila species, with greater than 90% of residues identical within the conserved sequences. a, CAATTA; b, TAATTA; c, AATTAT; d, AATTAC; e, ATTACA; f, GCAAATT; g, TTGCATA; h, GCTCATTA; Lz, RACCRCA; Sd, AAATATTT. (B) Expected and observed occurrences of each element. (128 KB PDF) [file pbio.0060125.sg006.pdf]

A

Gene	Location	a	b	c	d	e	f	g	Lz	Sd	Sequence
		Shared elements							500 bp		Conserved sequence
**Or10a**	ab1	■									CCGAATAACAAGTCAATTA
**Or42b**	ab1, L		■	■							TTAATTAAACATAACATAGCGCAGCAGAGAAATTCTCAAGTTAATAATTAAACAACAGCAAG
**Or92a**	ab1		■	■	■						TTAAGTAATTATGAGGTCAGCGAAGGGTGTCTCATGCGAATTTAAT
**Or59b**	ab2		■		■	■			■		TAATTACAG
**Or85a**	ab2										
**Or22a**	ab3							■	■		CTGCGGCATGCAGCTCATTAATAATACAGATAATGTGTTTGGCGGCAATCATTTTTCGGTTGCATGCCATCGCGGTGGCATTGATTAACACCGCCA
**Or22b**	ab3										
**Or85b**	ab3										GGCTATAAA
**Or56a**	ab4	■		■							AAACAGAGACAATTATTGGACAATTATTGGCACTTA
**Or7a**	ab4, L	■					■				CAAATATGCAATAACAAAATGCTGCTTGTGACTTATGTTATGTACAAATTAGCAATTAACAATTGACGATTCATTTAAATCATTCAGAGACCAAAATGCGAAAATAAAAAACTATTTTTTATAGAAAA
**Or47a**	ab5, L										GCGTGAATAAAGAACATTAAAATTTAACCGGAGTTCTCATTTACTATGCCTAAAGAGGTGTCAAACGGAGGTATTATTT
**Or82a**	ab5, L	■		■							TTACAATTATCGTGTCAGCAC
**Or49b**	ab6, L								■		AATGAAAAATATATTGAA
**Or67c**	ab7	■		■					■		AGTTAAAAGTTTAGGCACAATTATCAACACTTTTCAAGGCGCGACATGATGGGGCATAAAAACTTATCAGACACGCCC
**Or98a**	ab7										AAATTCAAAAT
**Or9a**	ab8		■		■	■					AAATCGTGTAATTAGAGCCATAAATGCACTTCAAGTCCACCCAGGTCCACACCC
**Or43b**	ab8										
**Or67b**	ab9, L		■	■					■		ACGCCCATCCAATTTGTATAATTATTTGTGTCTCCTATCGAGTTGCACAATAACCACAACGAATAGCAAATT
**Or69aA**	ab9										CTTCCGCCGGAAATGGAGTGCAGAACGAGCAGCACTTGTCTGCTGATATCAGCAGCCATAGACAAGCGCATAAAAGCCTGCTAACCAGCTCAAAGGCATCATCATTACGAGCTTGACACGAATATAAACTGAATAAACAAACAAATAATGTGCG
**Or69aB**	ab9		■	■	■						GACAGCCACGTTTTAGAGCAGGATGGAATTTGAGAACTACTTTAAGTATTTTGACAGCATTTTGCGTCTGGCGATGTTTCCACGAGGAAGATTTGTATCCAACGTTTCGAAGGGCATTCTTTTTGCTAGGTAACTTTTGCCTGGCTTACCAATGTATTGGAGTAATTATAGATTGTATAGATTGGT
**Or49a**	ab10	■	■				■				GGACAAAAGATCTGTATTGGGTTATTTGCATAAACGAGCCGCTGAAAATTTGTCATCTAATTAATTGGCTCGAATTTATTTAACATTTGTATTTAAATATTCATTG
**Or67a**	ab10								■		TTTTGGAAAGT
**Or85f**	ab10	■	■		■				■		AAGTTATAACCCGATTGGCCGTGGTAATTGCAACTCTGATTCCGCGCATCTCTCAATTGATCTTAATTAATGTTCATTT
**Or35a**	ac1,L	■				■					GGACAAGCCATTACATCGTAAATGAGGCAATATTAATGTGAGACTTTCAAATGTTTCCGTTTGGCTTGAAACTGGAGGATAATAACTTAATTGAGCCATTGCACGCATGGGAGACTTCATTTAAAAGTTGAGAAACTGTC
**Or13a**	ai1, L										CTTTCTAAAATATT
**Or1a**	L		■	■							CCTCGAGCGTATAATTAAACCATTTGCTAGCCGAACCAACCCGTTTCACCTCGTCAAGTT
**Or2a**	L										TCCCAGCCACTTAAGCGACAAGTTTGGCCA
**Or22c**	L	■									TAGACGTAAGCATAAAAACAATTAAAACTTTTGCCAAAGGCACAACTTGCTAACGCTGCACTGAAATTA
**Or24a**	L	■		■							AAACGTTGCCTGATTGGGGGTTAACAATTTGTAAAACTTATCGAACTATGAGCAAGGACAACGCCCAGCTCATCAATTATGCTGCTCAAGTGCTTTGTGTGATTCT
**Or30a**	L										ACAGACAAATTGCCACCGATTAAGATTTTTTATCGGAG
**Or33a**	L										AGTATAAAA
**Or45a**	L						■		■	■	GATGTTGCATACTTTCGTACTTTCATACTAACAAATTCCG
**Or45b**	L										GGTGGGCAAACGGGTTTTTTTTATTCGGGTTCTGCAACTGTTTTTTTTGGCGACTCGCTGACACGCTGGCCTCCTTTTTCCTCGCTTCCAGCACTGGCACTCCTAGCTTTTCCACTTGC
**Or59a**	L	■	■	■	■	■					TAATTACATAATTG
**Or63a**	L	■		■	■				■	■	GCAAAGTATTTCAATTATTTAGTGAAACTTTGCCCACGTAATTGGGCGTGG
**Or74a**	L	■									TGAATTTAATATTTCATCAGGCCGGCGGCAATTAAAATTAGAATATAAATGG
**Or83a**	L										AAGTGGCGTTATGACTCGTAGAGCCCAGTTGCCAAAC
**Or85c**	L										ATTGTTGAAAACTGAAAAGATTTAATTTATTTCAAGTGAGAGCGAAAGAAGACTTCACTTCGGTGGGATTAGACTTGGGTGTGATGCGCTGCTACCGATTCGCTTTCGAATTGCATTTATTCAAACGTTTCAATTGCTTTCCGTCGGATTCAATCTTCGAGTGATTTATTGTG
**Or94a**	L	■	■	■	■	■		■			TAACCAGCCAAAATATTTATACAACTAATGAGCAGGGCAATTACAATAATTA
**Or94b**	L	■	■	■	■	■		■		■	TAATTATTGTAATTGCCCTGCTCATTAGTTGTATAAATATTTTGGCTGGTTA
**Or98b**	L								■	■	GCAAAAGTTTTCACATTTGAAATGCAGCTAGGATTTGTTTTAGTTTTTGCTTAGCATTGCTGCCAAATATTTGCAGTGGCTCACAGATTTG
**Or33b**	L, ab 2,5			■							AAGAAAAGGTGTGAATTGGTCATAGCAAATTATAGG

B

Sequence	**a** CAATTA	**b** TAATTA	**c** AATTAT	**d** AATTAC	**e** ATTACA	**f** TTGCATA	**g** GCTCATTA
Expected	3.1	2.14	4.2	2.4	2.48	0.62	0.08
Observed	17	13	16	9	6	3	3
