# Supplementary material for: A Regulatory Code for Neuron-Specific Odor Receptor Expression
Source: PLoS Biol. 2008 May 27;6(5):e125. doi: 10.1371/journal.pbio.0060125 (PMC2430909; doi:10.1371/journal.pbio.0060125)
Supplement: Figure S7 — (A) Map of elements in the upstream sequences of axon-guidance genes (table adapted from [1]). The dots surrounding the 42a4 symbol indicate that a single iteration of the AGTGTAAA sequence is observed. (B) Sequence alignment of elements upstream of axon-guidance genes. The Oligo-1 element upstream of Ptp10D is not shown because although present in 5 species, its position is not well-conserved. (91 KB PDF) [file pbio.0060125.sg007.pdf]

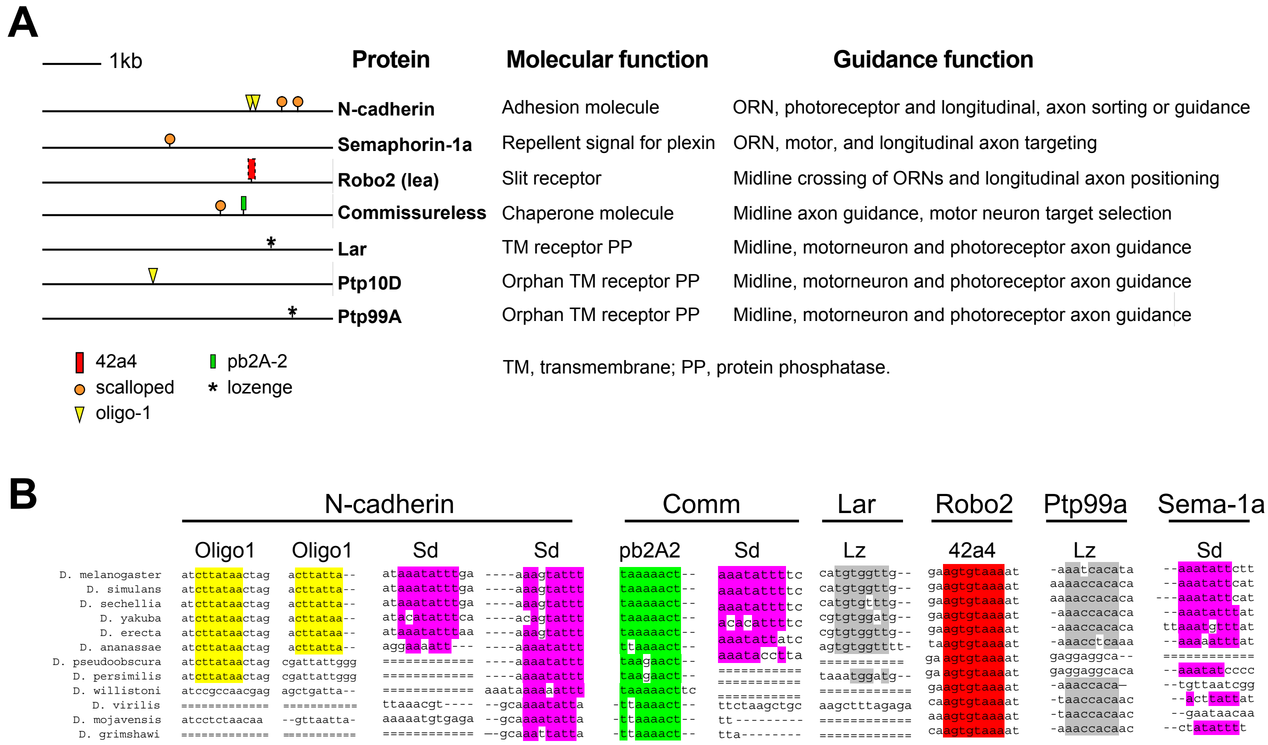
A
1kb
Protein
Molecular function
Guidance function
N-cadherin
Adhesion molecule
ORN, photoreceptor and longitudinal, axon sorting or guidance
Semaphorin-1a
Repellent signal for plexin
ORN, motor, and longitudinal axon targeting
Robo2 (lea)
Slit receptor
Midline crossing of ORNs and longitudinal axon positioning
Commissureless
Chaperone molecule
Midline axon guidance, motor neuron target selection
Lar
TM receptor PP
Midline, motorneuron and photoreceptor axon guidance
Ptp10D
Orphan TM receptor PP
Midline, motorneuron and photoreceptor axon guidance
Ptp99A
Orphan TM receptor PP
Midline, motorneuron and photoreceptor axon guidance
42a4
pb2A-2
scalloped
* lozenge
oligo-1
TM, transmembrane; PP, protein phosphatase.
B
N-cadherin
Comm
Lar
Robo2
Ptp99a
Sema-1a
Oligo1
Oligo1
Sd
Sd
pb2A2
Sd
Lz
42a4
Lz
Sd
D. melanogaster
D. simulans
D. sechellia
D. yakuba
D. erecta
D. ananassae
D. pseudoobscura
D. persimilis
D. willistoni
D. virilis
D. mojavensis
D. grimshawi
