# Supplementary material for: A Regulatory Code for Neuron-Specific Odor Receptor Expression
Source: PLoS Biol. 2008 May 27;6(5):e125. doi: 10.1371/journal.pbio.0060125 (PMC2430909; doi:10.1371/journal.pbio.0060125)
Supplement: Text S1 — (51 KB DOC) [file pbio.0060125.sd001.doc]

**Supplemental Information**

**Conserved *cis-­*elements upstream of larval and antennal *Or* genes**

In addition to the 7 maxillary palp receptors, the *Or* gene family contains 53 other members, of which 25 are expressed in the basiconic, coeloconic, or intermediate sensilla of the antenna, and 25 are expressed in the larval olfactory system; 8 of the 53 are expressed in both [5, 6, 12]. Using a comparative bioinformatic approach we performed a large-scale analysis of sequence conservation in the 500 bp upstream of each of these 42 *Or* genes across all 12 *Drosophila* species (Figure S5). We were able to identify one or more conserved sequences in the upstream DNA of 38 of the 42 *Or* genes (Figure S5). The sequences showing the highest conservation score for each individual gene are, by analogy to maxillary palp *Or* genes, strong candidates for regulatory regions and are listed in Figure S6a. Most of these sequences are long enough to accommodate binding sites for multiple regulatory proteins. In order to identify interesting elements within these sequences, we used the OLIGO-ANALYSIS program that can identify motifs shared among sequences [18]. In the analysis of maxillary palp *Or* genes described above we sought elements that are unique to each gene; here we sought elements that are highly conserved and shared among *Or* genes.

Results of this analysis indicated that many of the conserved regions are indeed modular in nature and consist of shorter motifs that are shared among subsets of receptor promoters (Figure S6a). We identified seven elements that are present upstream of at least three genes. All seven elements are present at higher frequencies in these conserved upstream regions than in 100 random sequences of the same length (Figure S6b); the random sequences had the same base pair composition as that of all *Drosophila* non-coding DNA. This analysis suggests that although each receptor gene may have a unique combination of *cis*-regulatory elements, some elements are shared by subsets of receptors as expected for a combinatorial code.

To examine further the combinatorial nature of the *cis*-regulatory code, we also searched for predicted binding sites of two transcription factors that have been shown to affect *Or* gene expression in *D. melanogaster,* Lozenge (Lz) [15] and Sd (Figure 5). In the 500 bp upstream region of each of the 42 *D. melanogaster* genes we found 10 *Or* genes with putative binding sites for Lz and four with putative sites for Sd that are conserved across many *Drosophila* species (Figure S6a).

**Axon guidance genes with conserved *Or* cis-regulatory elements**

We note with interest that some *Or* regulatory elements are located upstream of genes required for ORN axon targeting or sorting, at positions that have been conserved for tens of millions of years (Figure S7a). The 42a4 motif is found upstream of the *robo2* gene, which is required for normal midline crossing of ORN axons [10]. The site has been conserved in all 12 species, although a single iteration of the AGTGTAAA sequence is observed, rather than two in an inverted repeat (Figure S7b). Both Sd and Oligo-1 [15] binding sites are found upstream of *N-cadherin* (Figure S7a,b), which plays a role in ORN axon sorting [9]. The *Semaphorin-1a* gene, required for ORN axon targeting [11, 13, 17], contains an element that matches a Sd binding site at 7 of 8 positions. *Or* regulatory elements are also found upstream of four other genes that have not been tested for a role in axon guidance in the olfactory system, but act in guidance of photoreceptor, midline, or motorneuron axons [1](Figure S7a,b). The *Commissureless* gene contains the ORN-specific element pb2A-2 [15] in all 12 species at the same upstream position (Figure S7a), and a Sd binding site. Three other axon-guidance proteins, Ptp99A, Lar, and Ptp10D, [4] contain Lz or Oligo-1 sites [15]. Ptp10D is expressed in the antenna lobe [14].

1. Araujo SJ, Tear G (2003) Axon guidance mechanisms and molecules: lessons from invertebrates. Nat Rev Neurosci 4: 910-22

2. Blanchette M, Kent WJ, Riemer C, Elnitski L, Smit AF, Roskin KM, Baertsch R, Rosenbloom K, Clawson H, Green ED, Haussler D, Miller W (2004) Aligning multiple genomic sequences with the threaded blockset aligner. Genome Res 14: 708-15

3. Chiaromonte F, Yap VB, Miller W (2002) Scoring pairwise genomic sequence alignments. Pac Symp Biocomput: 115-26

4. Clandinin TR, Lee CH, Herman T, Lee RC, Yang AY, Ovasapyan S, Zipursky SL (2001) Drosophila LAR regulates R1-R6 and R7 target specificity in the visual system. Neuron 32: 237-48

5. Couto A, Alenius M, Dickson BJ (2005) Molecular, anatomical, and functional organization of the Drosophila olfactory system. Curr Biol 15: 1535-47

6. Fishilevich E, Domingos AI, Asahina K, Naef F, Vosshall LB, Louis M (2005) Chemotaxis behavior mediated by single larval olfactory neurons in Drosophila. Curr Biol 15: 2086-96

7. Fishilevich E, Vosshall LB (2005) Genetic and functional subdivision of the Drosophila antennal lobe. Curr Biol 15: 1548-53

8. Hallem EA, Ho MG, Carlson JR (2004) The molecular basis of odor coding in the Drosophila antenna. Cell 117: 965-79

9. Hummel T, Zipursky SL (2004) Afferent induction of olfactory glomeruli requires N-cadherin. Neuron 42: 77-88

10. Jhaveri D, Saharan S, Sen A, Rodrigues V (2004) Positioning sensory terminals in the olfactory lobe of Drosophila by Robo signaling. Development 131: 1903-12

11. Komiyama T, Sweeney LB, Schuldiner O, Garcia KC, Luo L (2007) Graded expression of semaphorin-1a cell-autonomously directs dendritic targeting of olfactory projection neurons. Cell 128: 399-410

12. Kreher SA, Kwon JY, Carlson JR (2005) The molecular basis of odor coding in the Drosophila larva. Neuron 46: 445-56

13. Lattemann M, Zierau A, Schulte C, Seidl S, Kuhlmann B, Hummel T (2007) Semaphorin-1a controls receptor neuron-specific axonal convergence in the primary olfactory center of Drosophila. Neuron 53: 169-84

14. Qian M, Pan G, Sun L, Feng C, Xie Z, Tully T, Zhong Y (2007) Receptor-like tyrosine phosphatase PTP10D is required for long-term memory in Drosophila. J Neurosci 27: 4396-402

15. Ray A, van der Goes van Naters W, Shiraiwa T, Carlson JR (2007) Mechanisms of odor receptor gene choice in Drosophila. Neuron 53: 353-69

16. Siepel A, Bejerano G, Pedersen JS, Hinrichs AS, Hou M, Rosenbloom K, Clawson H, Spieth J, Hillier LW, Richards S, Weinstock GM, Wilson RK, Gibbs RA, Kent WJ, Miller W, Haussler D (2005) Evolutionarily conserved elements in vertebrate, insect, worm, and yeast genomes. Genome Res 15: 1034-50

17. Sweeney LB, Couto A, Chou YH, Berdnik D, Dickson BJ, Luo L, Komiyama T (2007) Temporal target restriction of olfactory receptor neurons by Semaphorin-1a/PlexinA-mediated axon-axon interactions. Neuron 53: 185-200

18. van Helden J, Andre B, Collado-Vides J (1998) Extracting regulatory sites from the upstream regions of yeast genes by computational analysis of oligonucleotide frequencies. J. Mol. Biol. 281: 827-842

19. Yao CA, Ignell R, Carlson JR (2005) Chemosensory coding by neurons in the coeloconic sensilla of the Drosophila antenna. J Neurosci 25: 8359-67
